# TLR-9 agonist and CD40-targeting vaccination induces HIV-1 envelope-specific B cells with a diversified immunoglobulin repertoire in humanized mice

**DOI:** 10.1371/journal.ppat.1009025

**Published:** 2020-11-30

**Authors:** Véronique Godot, Colas Tcherakian, Laurine Gil, Iñaki Cervera-Marzal, Guangming Li, Liang Cheng, Nicolas Ortonne, Jean-Daniel Lelièvre, Giuseppe Pantaleo, Craig Fenwick, Mireille Centlivre, Hugo Mouquet, Sylvain Cardinaud, Sandra M. Zurawski, Gerard Zurawski, Pierre Milpied, Lishan Su, Yves Lévy

**Affiliations:** 1 Vaccine Research Institute, Creteil, France; 2 Inserm U955, Equipe 16, Créteil, France; 3 Hôpital Foch, Service de Pneumologie, Suresnes, France; 4 Aix Marseille Université, CNRS, INSERM, Centre d'Immunologie de Marseille-Luminy, Marseille, France; 5 Lineberger Comprehensive Cancer Center, University of North Carolina at Chapel Hill, Chapel Hill, North Carolina, United States of America; 6 Department of Microbiology and Immunology, University of North Carolina at Chapel Hill, Chapel Hill, North Carolina, United States of America; 7 AP-HP, Hôpital Henri-Mondor Albert-Chenevier, Service d’Anatomopathologie, Créteil, France; 8 AP-HP, Hôpital Henri-Mondor Albert-Chenevier, Service d’Immunologie Clinique et Maladies Infectieuses, Créteil, France; 9 Service of Immunology and Allergy Lausanne University Hospital, Lausanne, Switzerland; 10 Swiss Vaccine Research Institute, Lausanne University Hospital, University of Lausanne, Lausanne, Switzerland; 11 Laboratory of Immunology, Department of Immunology, Institut Pasteur, Paris, France; 12 INSERM U1222, Paris, France; 13 Baylor Scott and White Research Institute and INSERM U955, Dallas, Texas, United States of America; Emory University, UNITED STATES

## Abstract

The development of HIV-1 vaccines is challenged by the lack of relevant models to accurately induce human B- and T-cell responses in lymphoid organs. In humanized mice reconstituted with human hematopoietic stem cells (hu-mice), human B cell-development and function are impaired and cells fail to efficiently transition from IgM B cells to IgG B cells. Here, we found that CD40-targeted vaccination combined with CpG-B adjuvant overcomes the usual defect of human B-cell switch and maturation in hu-mice. We further dissected hu-B cell responses directed against the HIV-1 Env protein elicited by targeting Env gp140 clade C to the CD40 receptor of antigen-presenting cells. The anti-CD40.Env gp140 vaccine was injected with CpG-B in a homologous prime/boost regimen or as a boost of a NYVAC-KC pox vector encoding Env gp140 clade C. Both regimens elicited Env-specific IgG-switched memory hu-B cells at a greater magnitude in hu-mice primed with NYVAC-KC. Single-cell RNA-seq analysis showed gp140-specific hu-B cells to express polyclonal IgG1 and IgG3 isotypes and a broad Ig VH/VL repertoire, with predominant VH3 family gene usage. These cells exhibited a higher rate of somatic hypermutation than the non-specific IgG^+^ hu-B-cell counterpart. Both vaccine regimens induced splenic GC-like structures containing hu-B and hu-Tfh-like cells expressing PD-1 and BCL-6. We confirmed in this model that circulating ICOS^+^ memory hu-Tfh cells correlated with the magnitude of gp140-specific B-cell responses. Finally, the NYVAC-KC heterologous prime led to a more diverse clonal expansion of specific hu-B cells. Thus, this study shows that CD40-targeted vaccination induces human IgG production in hu-mice and provides insights for the development of a CD40-targeting vaccine to prevent HIV-1 infection in humans.

## Author summary

Mice reconstituted with human hematopoietic stem cells (hu-mice) are a powerful tool for the study of human immune function *in vivo* and can be useful as a pre-clinical model to rank vaccination strategies. However, in hu-mice, human B cell-development and function are impaired and cells fail to efficiently transition from IgM B cells to IgG B cells. One finding of our study is that CD40-targeted vaccination combined with CpG-B adjuvant overcomes the usual defect of human B-cell switch and maturation in hu-mouse models. We further reported that the HIV-1 envelope-specific IgG^+^ hu-B cells elicited in hu-mice by the anti-CD40.Env vaccine used more VH3 and VH4 family genes and displayed higher rates of somatic hypermutations than the non-specific IgG^+^ hu-B-cell counterpart. VH3 antibodies are essential for antiviral immunity. We also showed that monitoring ICOS^+^ circulating Tfh cells seven days after the last booster immunization is a surrogate marker for vaccine-induced HIV-1-specific B-cell responses. Overall, we report important results, both in the setting of this hu-mouse model and for a prophylactic HIV vaccine.

## Introduction

Challenges in the development of HIV-1 vaccines include more accurately directing protective immune responses. Developing appropriate pre-clinical animal models will help to further this aim. Research on preventive HIV-1 vaccines first explored the efficacy of B cell-based vaccines using a bivalent recombinant envelope (Env) gp120 protein as a unique immunogen in a homologous prime/boost vaccination regimen in healthy volunteers [1,2]. Despite the induction of HIV-1 Env-specific antibodies in all vaccinated individuals, such vaccination strategies failed to prevent HIV-1 infection or delay disease progression and led to the search for prophylactic vaccination strategies towards T cell-based vaccines. The basic rationale for eliciting T-cell responses is to control the early viral load after infection and limit HIV-1 expansion/spreading after transmission. However, T-cell responses elicited by adenoviral (rAd5)-based vaccines, either used in homologous or heterologous (DNA prime/ rAd5 boost) vaccine regimens, have not fulfilled their protective function in humans [3–5]. The RV144 Phase 3 vaccine trial evaluated priming with the Alvac-HIV vector combined with a protein boost using a bivalent recombinant gp120 protein in healthy volunteers [6]. Intriguingly, partial protection of vaccinated individuals was achieved, with the generation of non-neutralizing antibodies directed against the HIV-1 Env V1V2 region [7–10]. Correlates of protection also included the generation of Env-specific T-cell responses and showed the importance of IgG isotype and ADCC (antibody-dependent cell-mediated cytotoxicity) of the antibody responses [7–10]. Overall, correlative results of the RV144 trial support the usefulness of HIV-1 Env proteins for the design of preventive vaccines that induce both B- and T-cell responses [6]. However, the rate of protection in the RV144 trial rapidly waned and underscores the need to improve the durability of such protective responses.

These observations indicate that better vaccine design and changes in the immunization regimens must be explored for the development of effective vaccination strategies. Following the RV144 trial, several Phase 2b/3 clinical trials were launched to test various combinations of viral or DNA vectors with Env protein in heterologous prime/boost strategies [11–13]. In recent years, efforts have been made to improve the stability, conformation, and immunogenicity of the Env protein used in preventive vaccines, as well as their delivery to the immune system [13,14]. Our group and others have focused on a strategy to target vaccine antigens to receptors (DEC-205, CD40, DCIR, LOX-1, Langerin) on dendritic cells (DCs), with the aim of improving antigen presentation and activation of antigen-specific immune responses [15–21]. We have reported that the delivery of HIV antigens through these receptors, even in minute amounts, induces strong and sustained T- and B-cell responses *in vitro* and *in vivo* in animal models, including non-human primates (NHPs) and humanized mice reconstituted with human hematopoietic stem cells (hu-mice) [15–21]. Among these candidate vaccines, we have developed an anti-human CD40 antibody (recombinant human IgG4) fused to a string of HIV-1 T-cell epitopes (derived from Gag, Pol and Nef) that elicits strong and polyfunctional T-cell responses in NHPs [16,20] or hu-mice [21,22]. In vaccinated hu-mice, these T-cell responses were associated with a decrease of the viral reservoir that correlated with a significant control of HIV-1 replication [21]. The anti-human CD40 antibody fused via a flexible linker to the heavy (H)-chain C terminus to codon-optimized Env gp140 protein from the clade C HIV-1 96ZM651 (called ZM96) strain has been tested in NHPs primed with a replication-competent NYVAC-KC vaccinia virus vector encoding HIV-1 Gag, Nef, Pol, and Env gp140 sequences [16]. The CD40 targeting vaccine provided an advantage, in the magnitude and breadth of the antibody response, in raising antibodies with ADCC properties and in improving the durability of antibody responses [16].

Here, we sought to investigate the mechanism and contribution of Tfh cells to B-cell activation and Ig repertoire diversification in depth using hu-mice immunized with the anti-CD40.Env gp140 candidate vaccine, either in a homologous prime/boost regimen or for boosting immunity primed by the NYVAC-KC vaccine.

## Results

### TLR9 stimulation by CpG and CD40-targeting vaccination cooperatively induces B-cell maturation and the emergence of IgG^+^ B cells, increases CD40 expression on human antigen-presenting cells, and expansion of memory T cells in vaccinated hu-mice

**Fig 1:**
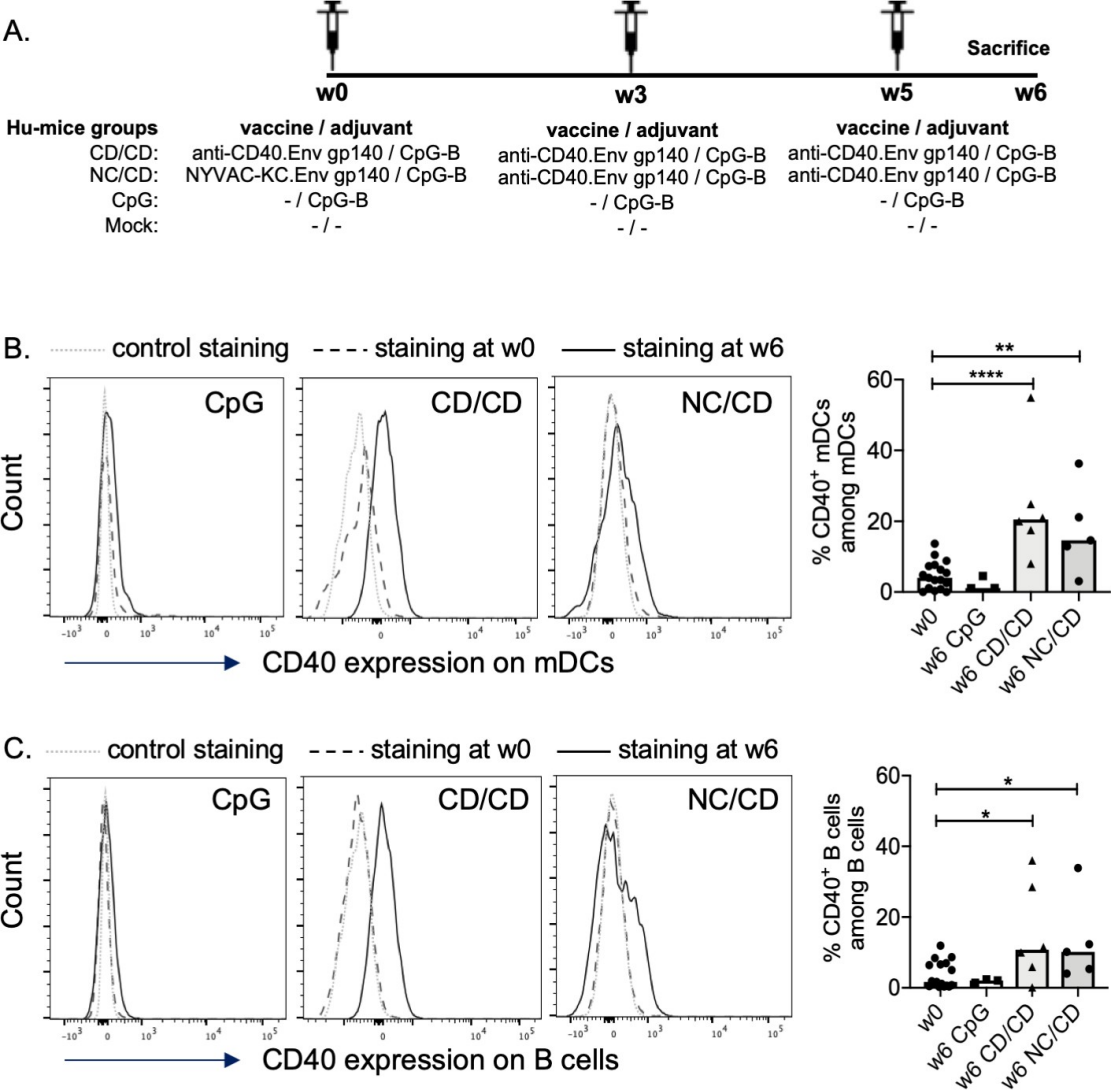
Enhanced expression of CD40 on myeloid (m) human DCs and B cells in vaccinated hu-mice. **(A)** Schematic representation of the experimental design. (**B-C)** Representative histograms and summary data show the frequency of human blood CD40^+^ mDCs (**B**) and human CD40^+^ B lymphocytes (**C**) in the different vaccination settings. See the gating strategy of human mDCs and B cells in the S1 Fig. Individual values are presented, along with the median. The values of all hu-mice at week 0 was used as the base values. Mann-Whitney *U*-tests were used for comparisons. *p < 0.05, **p < 0.01, ****p<0.0001.

We and others have shown that a functional human immune system that includes human DCs and T and B cells is developed in immunodeficient mice transplanted with human fetal liver-derived CD34^+^ cells. We recently reported that such hu-mice develop functional and potent human T-cell responses that can significantly reduce the HIV-1 reservoir when HIV-T cell epitopes are targeted to the CD40 receptor [21]. Here, we tested whether such hu-mice, with efficient human immune-cell reconstitution (S1, S2A and S2B Figs), can intiate B-cell immunity in response to CD40 targeting Env gp140 protein from the clade C HIV-1 ZM96 (anti-CD40.Env gp140 called CD) adjuvanted with a TLR-9 agonist (CpG). CpG/CD was administered either alone at weeks 0, 3, and 5 (CD/CD group) or at weeks 3 and 5, following a prime at week 0 with a recombinant replication competent NYVAC-KC vector encoding the homologous Env gp140 clade C ZM96 (NC) (NC/CD group) (Fig 1A).

The number of human splenic CD45^+^ cells was significantly higher in vaccinated hu-mice than in control animals that received either PBS or CpG alone (S2C Fig). The expression of CD40 on human DC and B cells increased significantly in both CpG/CD vaccinated groups by week 6 (one week following the last boost) relative to baseline (week 0) (Fig 1B and 1C), which may have contributed to the vaccine effects.

**Fig 2:**
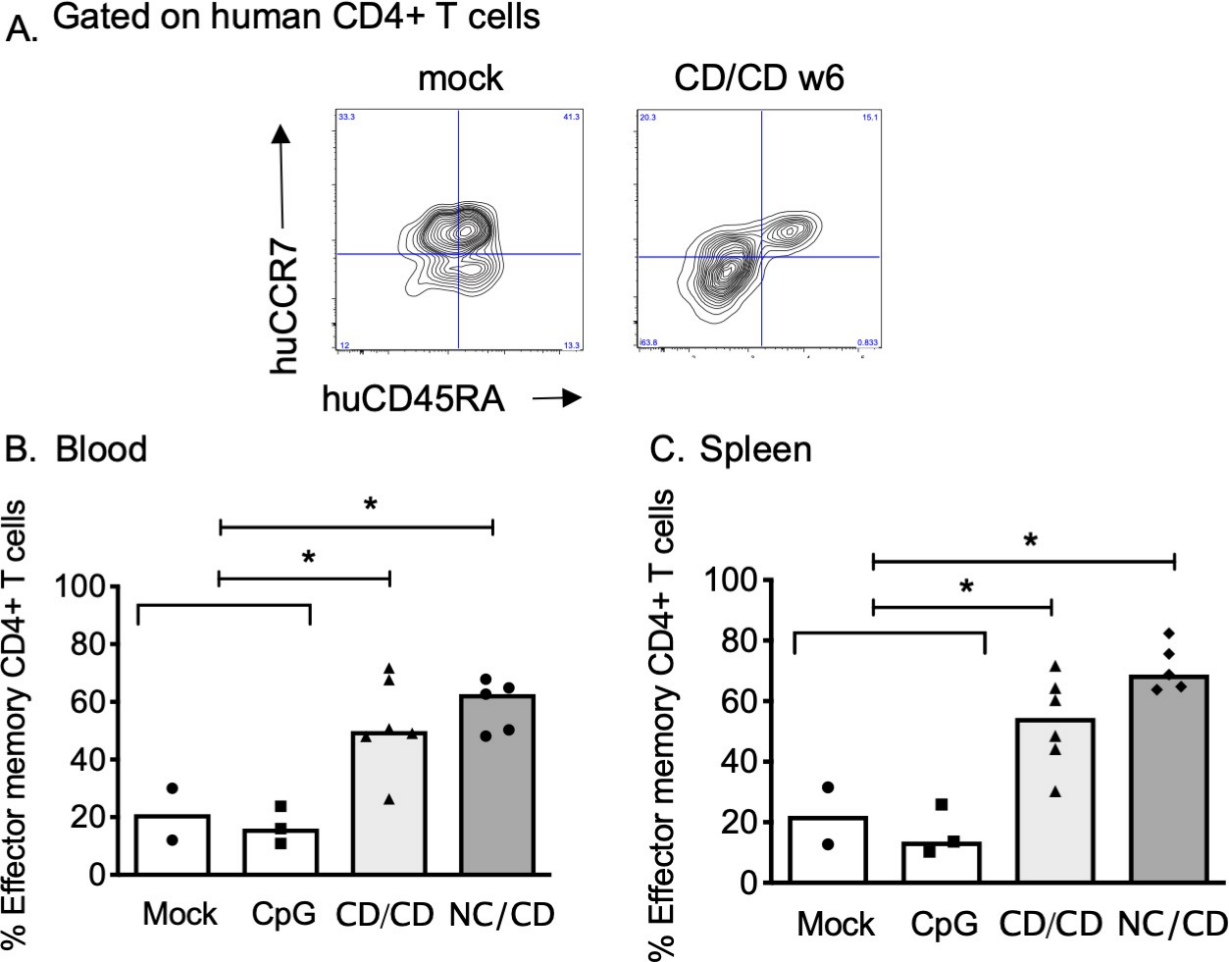
Vaccination elicits expansion of human memory T cells. **(A)** Flow cytometry of splenocytes from hu-mice injected three times with PBS (mock) or the anti-CD40.Env gp140 vaccine (CD/CD) one week after the last injection (w6). The human CD4^+^ T cells (see their gating strategy in S1 Fig) were represented in a huCCR7 *versus* huCD45RA dot blot to identify the human effector memory CD4^+^ T cells (CCR7^-^ CD45RA^-^). (**B-C**) Frequency of human effector memory CD4^+^ T cells in the blood (**B**) and spleens (**C**) of hu-mice. Individual values are presented, along with the median. Mann-Whitney *U*-tests were used for comparisons between immunized and non-immunized hu-mice. *p < 0.05.

We next investigated whether vaccination of hu-mice induced human memory CD4^+^ T and B cells. We assessed the frequency of blood and spleen effector memory CD4^+^ hu-T cells (huCD45^+^CD4^+^CCR7^-^CD45RA^-^) (Fig 2A) and IgG-switched CD27^+^ memory hu-B cells (S3A Fig) by flow cytometry at week 6. Both the homologous (CD/CD) and heterologous (NC/CD) prime/boost vaccinations significantly increased the pool of effector memory CD4^+^ hu-T cells in the blood and spleen (Fig 2B and 2C). Similarly, we observed an increased number of IgG^+^ memory hu-B-cells in the blood and spleen in both vaccinated groups (S3B and S3C Fig).

### CpG/CD vaccination strategies elicit HIV-1 Env-specific IgG^+^ hu-B cells

**Fig 3:**
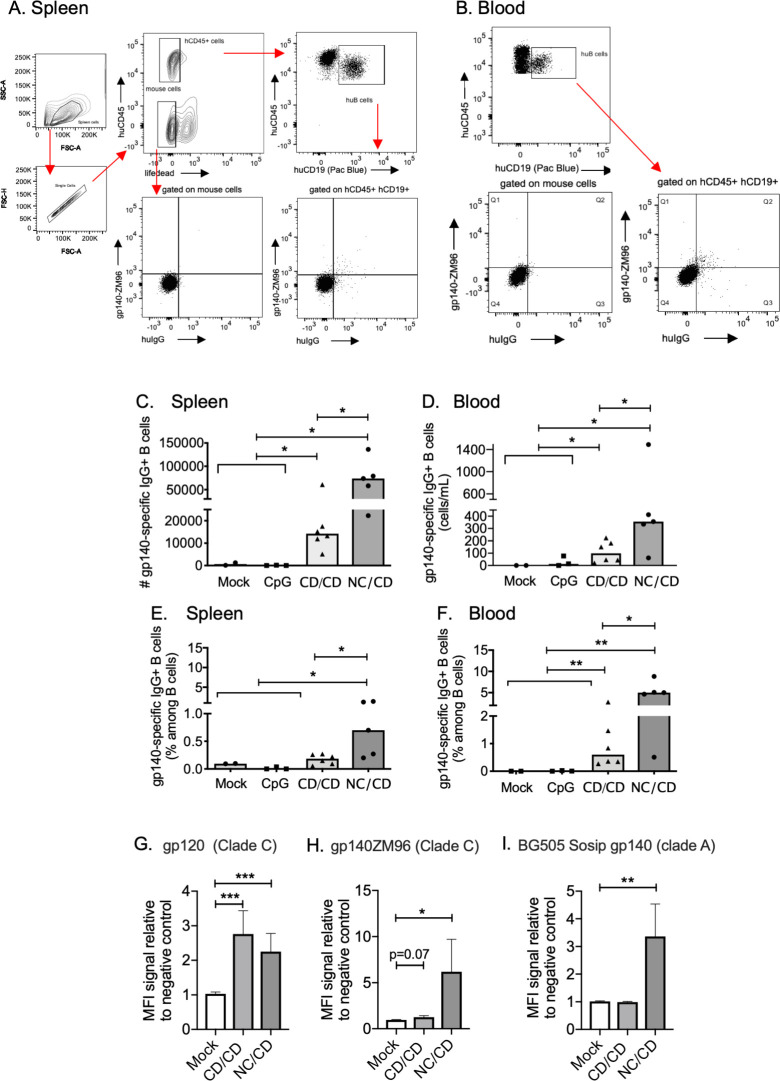
The CD/CD and NC/CD vaccination regimens elicit gp140ZM96 (Env)-specific human B cells. **(A-B)** Gating strategy to identify the gp140ZM96-specific IgG^+^ human B cells (called gp140^+^). (**A**) Flow cytometry of splenocytes from concatenated NC/CD hu-mice cells one week after the last injection (w6). After gating for single cells, viable cells within the huCD45^+^ gate were represented in a huCD45 *versus* huCD19 dot blot. Then hu-B cells were represented in a gp140ZM96 *versus* huIgG dot blot. The control staining for gp140ZM96 and huIgG are showed by gating on mouse cells. (**B**) The same gating strategy was used to identify the blood gp140^+^-specific IgG^+^ human B cells from concatenated NC/CD hu-mice cells one week after the last injection (w6). **(C-D)** Total number of gp140^+^ IgG^+^ human B cells in the spleens **(C)** and blood **(D)** of hu-mice. (**E-F**) Frequencies of gp140^+^ IgG^+^ human B cells in the spleens **(E)** and blood **(F)** of hu-mice. (C to F) Individual values are presented, along with the median. (**G to I**) Plasma HIV-1-specific human antibodies from hu-mouse samples evaluated for HIV-1 gp120 (**G**), gp140ZM96 (**H**) and BG505 SOSIP gp140 (**I**) binding by a custom multiplex assay (mean +/- sem). Three independent determinations were performed in triplicate. Mann-Whitney *U*-tests were used for comparisons. *p < 0.05, **p<0.01, ***p<0.001.

We next assessed the frequency of gp140-specific IgG^+^ memory hu-B cells in the blood and spleen of hu-mice at 6 weeks by flow cytometry using labeled gp140ZM96 (Fig 3A and 3B). The total number of gp140ZM96-specific IgG^+^ hu-B cells (called Env-specific B cells) was significantly higher in both compartments of the vaccinated hu-mice than those in the control mice (Fig 3C and 3D), together with a higher frequency of these specific-switched B cells at least in the blood (Fig 3E and 3F). These increases were significantly higher when the hu-mice were primed with NYVAC-KC (NC/CD group) relative to those of the CD/CD group: 356 [198–951] *versus* 99 cells/mL [38–193] in the blood (*P* = 0.03) and 736,000 cells [402,200–136,400] *versus* 142,100 cells [10,230–28,300] in the spleen (*P* = 0.017).

The plasma HIV-1-specific human antibodies from hu-mouse samples were then measured for HIV-1 Env binding by a custom multiplex assay. We found that plasma from CD/CD and NC/CD immunized hu-mice bound equally the gp120 clade C protein (Fig 3G). A higher level of gp140ZM96-binding human antibodies was observed in the NC/CD animals (Fig 3H). In addition, the NC/CD vaccination has generated antibodies cross-reacting with the gp140 clade A protein (Fig 3I).

To determine whether the CD40 targeting vaccination confers an advantage in terms of elicited humoral responses compared to the non-targeting vaccination, we immunized NRG hu-mice with an IgG4 fused via its C-terminal Fc-domains to the gp140ZM96 (IgG4.gp140ZM96 control construction) in combination with CpG. The timing of the injections was similar to the CD40 targeting vaccination regimen. We assessed at w6 the frequency of gp140-specific IgG^+^ B cells in the blood. The results showed in the S4 Fig confirmed that the CD40 targeting vaccination induces stronger specific humoral immune responses.

### Development of Env-specific hu-B cells correlates with the expansion of ICOS^+^ hu-Tfh cells

**Fig 4:**
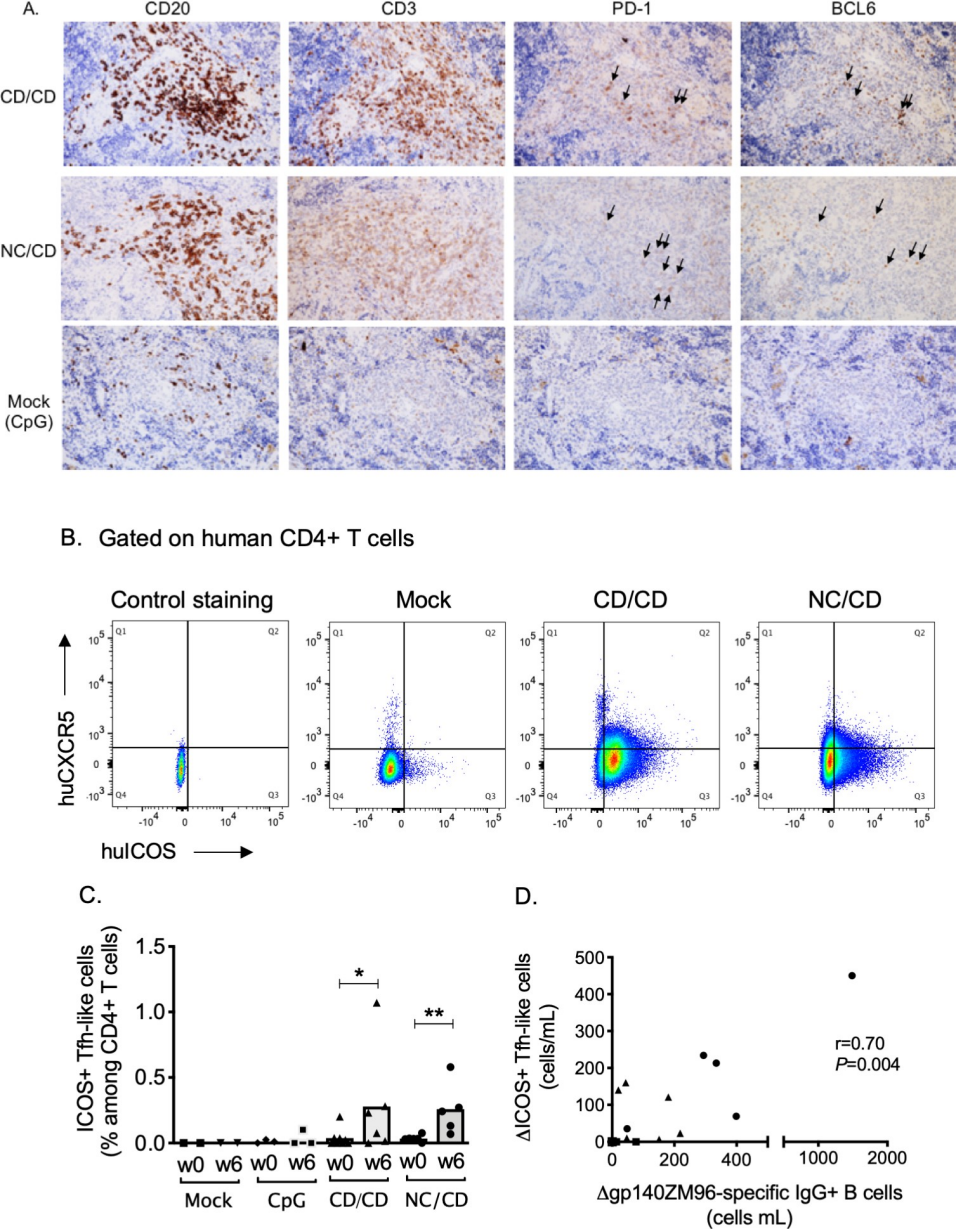
The CD/CD and NC/CD vaccination regimens elicit human memory Tfh cells that correlate with the expansion of gp140-specific human B cells. (**A**) Representative immunohistochemistry images of spleen sections from vaccinated hu-mice (NC/CD and CD/CD groups) and control animals that received CpG-B only (mock group). **(B)** Gating strategy for the evaluation of circulating human memory Tfh cells. Dot blots show concatenated data from mock hu-mice (n = 5), CD/CD (n = 6) or NC/CD (n = 5) vaccinated hu-mice one week after the last injection (w6). The human CD4^+^ T cells (see their gating strategy in the S1 Fig) were represented in a huCXCR5 *versus* huICOS dot blot to identify the subset of human memory blood ICOS^+^ Tfh cells. Control of hCXCR5 and hICOS staining was obtained by gating on mouse cells. **(C)** Frequency of human ICOS^+^ CXCR5^+^ Tfh cells in the blood of hu-mice. Individual values are presented, along with the median. Mann-Whitney *U*-tests were used for comparisons between w0 and w6 values obtained in immunized hu-mice. *p < 0.05, **p < 0.01. (**D)** Correlation between the increase in blood gp140-specific IgG^+^ hu-B cells (*x* axis) and the increase in blood ICOS^+^CXCR5^+^ hu-Tfh cells; hu-mice from the NC/CD group are represented as circles, hu-mice from the CD/CD group as triangles, and control groups as squares. Spearman’s rank test was used to assess correlations.

Germinal center (GC) Tfh cells in lymphoid tissues, such as the spleen and lymph nodes, play a key role in the development of class-switched memory B cells [23]. Tfh cells located within mature GCs express the transcription factor BCL6 and surface markers, including CXCR5, PD-1, and ICOS [23]. We therefore performed immunohistochemistry on spleens from hu-mice for human GC Tfh cells. Staining with anti-CD3 and anti-CD20 antibodies revealed a higher number of human T and B cells in the spleens of vaccinated animals than those of control hu-mice that received only CpG (Fig 4A). Human T and B cells were organized into GC-like structures in both the homologous CD/CD and heterologous NC/CD groups, with positive BCL6 and PD-1 staining (Fig 4A). Such GC-like structures were not detected in the spleens of control animals (Fig 4A). These results are consistent with the induction of Env-specific IgG^+^ hu-B cells observed only in the fully vaccinated hu-mice (Fig 3C–3F).

Mature Tfh cells in GCs can exit lymphoid organs into the blood and give rise to various subsets of circulating Tfh cells [24,25]. In humans, circulating ICOS^+^ Tfh cells are a subset of memory Tfh cells, which increases in the blood early after recall vaccination and is associated with protective antibody responses generated by memory B cells [26]. We used the same phenotypic approach to evaluate vaccine induction of circulating hu-Tfh cells by monitoring blood ICOS^+^ hu-Tfh cells at baseline and week 6, one week after the last boost of vaccine (Fig 4B). The frequency of blood ICOS^+^ hu-Tfh cells were significantly higher in hu-mice in both vaccine groups than in the controls (Fig 4C). The emergence of circulating ICOS^+^ hu-Tfh cells induced by the vaccination positively correlated with the increase in Env-specific IgG^+^ hu-B cells (Fig 4D), suggesting that human Tfh cells induced by the anti-CD40.Env gp140 vaccine contributed to the generation of the specific human B cell responses.

### Single-cell RNA-Seq analysis of Env-specific IgG^+^ hu-B cells reveals a high frequency of antibody-secreting human cells and GC hu-B cells

We next performed single-cell cloning from the spleens of hu-mice immunized with CD/CD and NC/CD for Env-specific IgG^+^ hu-B cells (anti-gp140^+^ B cells) and non-specific IgG^+^ hu-B cells (anti-gp140^-^ B cells) as controls (S5A and S5B Fig). Only the two vaccinated groups of hu-mice had a sufficient number of class-switched memory hu-B cells to perform scRNAseq analyses. In total, 877 IgG^+^ human B cells were analyzed. On average, 1,800 genes were expressed per single cell. Analyses of the quality of cells according to predefined criteria (see “Materials and Methods” and [27,28]) to detect and remove low-quality cells (*i*.*e*., a low number of transcripts, a high frequency of mitochondrial transcripts, or incomplete reconstruction of the IgG BCR heavy and light chain sequences) resulted in the overall exclusion of 216/318 cells from the CD/CD group and 398/559 from the NC/CD group. The remaining high-quality anti-gp140^+^ (n = 190) and anti-gp140^-^ IgG^+^ hu-B cells (n = 73) clustered into five distinct subsets corresponding to human plasmablasts, plasma cells, memory B cells, activated memory B cells, and GC B cells, based on their gene expression profiles (S6A Fig). Further analysis of marker genes related to B cells (*CD79A*, *HLA-DRA*, *CD19*, *MS4A1*), memory B cells (*SELL*, *FAIM3*, *CCR7*, *PARP15*), B cell activation (*CD40*, *CD86*, *CD69*, *AICDA*), GC B cells (*TCL1A*, *CD81*, *ELL3*, *IL4R*), the cell cycle (*STMN1*, *MKI67*, *MCM7*, *CCNB1*), and antibody-producing cells (*XBP1*, *FKBP11*, *PRDM1*, *SDC1*) confirmed the identity of the five distinct subsets of IgG^+^ hu-B cells (S6B Fig). In total, analysis of gp140^+^ and gp140^-^ IgG^+^ hu-B clones did not show significant differences in the distribution of the five B-cell subsets (S6C Fig), nor between the two regimens of vaccination (CD/CD & NC/CD, *P*>0.05 two-sided Chi-Square test), yielding a vast majority of IgG-secreting hu-B cells (S6C Fig) and a small fraction of GC B cells. These results, together with the induction of human memory Tfh cells, support the development of functional GC responses in vaccinated hu-mice.

### Heavy and Light Chain usage and VH repertoire diversity of Env-specific IgG^+^ hu-B cells from vaccinated hu-mice

**Fig 5:**
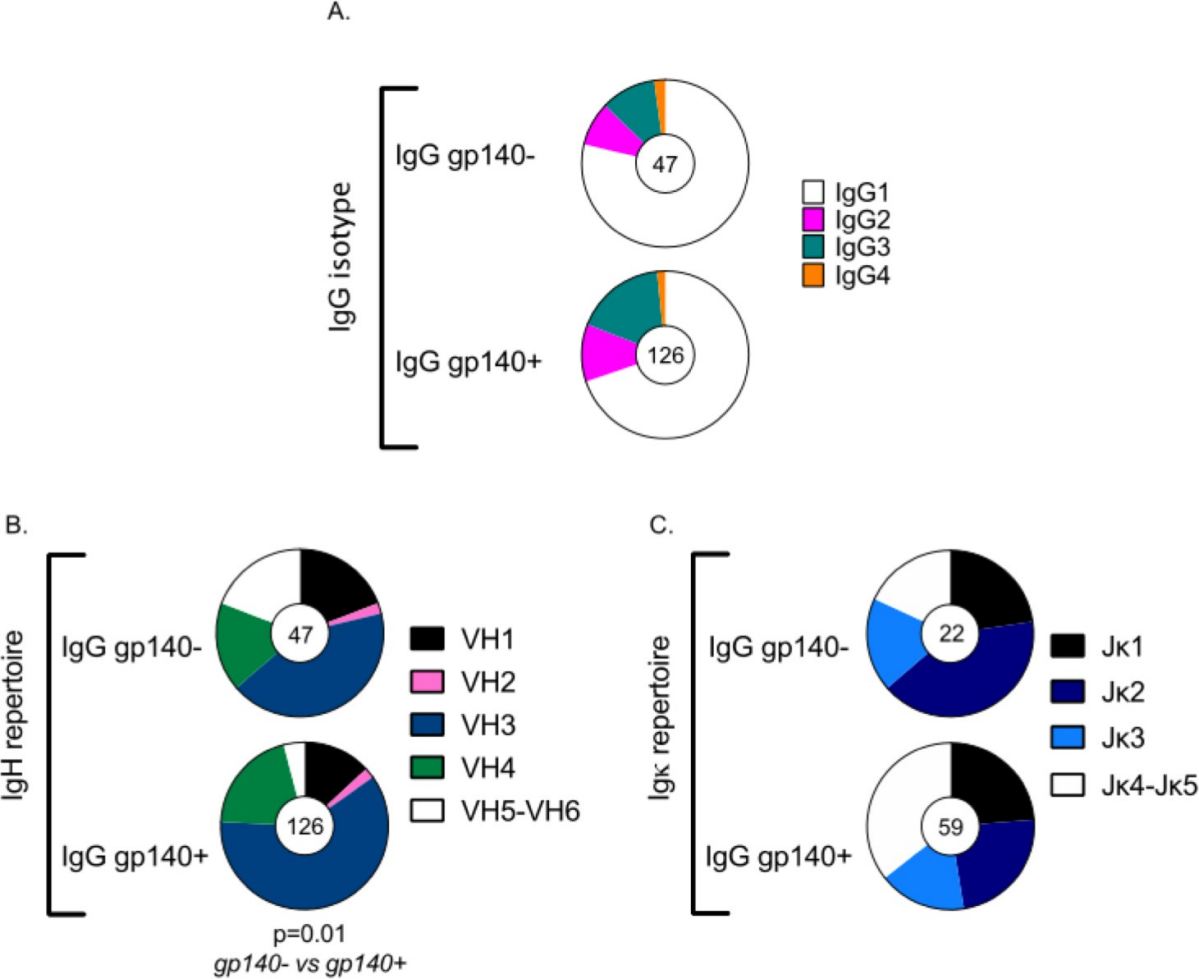
Immunoglobulin gene (IgH and IgL) repertoire of human gp140^+^ and gp140^-^ IgG^+^ B cells in vaccinated hu-mice. **(A)** Distribution of IgG subtypes within gp140^+^ and gp140^-^ IgG^+^ human B cells in all immunized hu-mice. (**B**) VH usage of the IgH and (**C**) Jk usage of the IgL in gp140^+^ and gp140^-^ IgG^+^ hu-B cells for all immunized hu-mice. The number of analyzed cells is indicated in the center of the pie charts. Distributions were compared using Chi-Square tests. The analysis of gene segment usage was carried out using NCBI IgBLAST software (http://www.ncbi.nlm.nih.gov/igblast/).

Analysis of IgG subclasses of gp140^+^ and gp140^-^ IgG^+^ hu-B cells showed a predominance of the IgG1 subclass in both populations (Fig 5A), whereas there was a greater proportion of IgG2 and IgG3 B cells among the gp140^+^ hu-B cells than the non-specific gp140^-^ IgG^+^ hu-B cells (Fig 5A).

Comparative analyses of IgH variable (V) and IgL joining (J) gene usage showed differences between gp140^+^-specific and non-specific IgG^+^ hu-B cells. The global distribution of VH gene usage differed between gp140^+^ and gp140^-^ IgG^+^ hu-B cells (*P* = 0.01, two-sided Chi-Square test), with greater usage of the VH3 gene family by the gp140^+^ hu-B cells (Fig 5B). Concerning light chain gene usage, there was a trend towards increased usage of the J(k)4/5 families in gp140^+^ specific hu-B cells (Fig 5C). These characteristics of the gp140^+^ IgG hu-B cells were found to be similar between both vaccine regimens (*P*>0.05 two-sided Chi-Square test).

**Fig 6:**
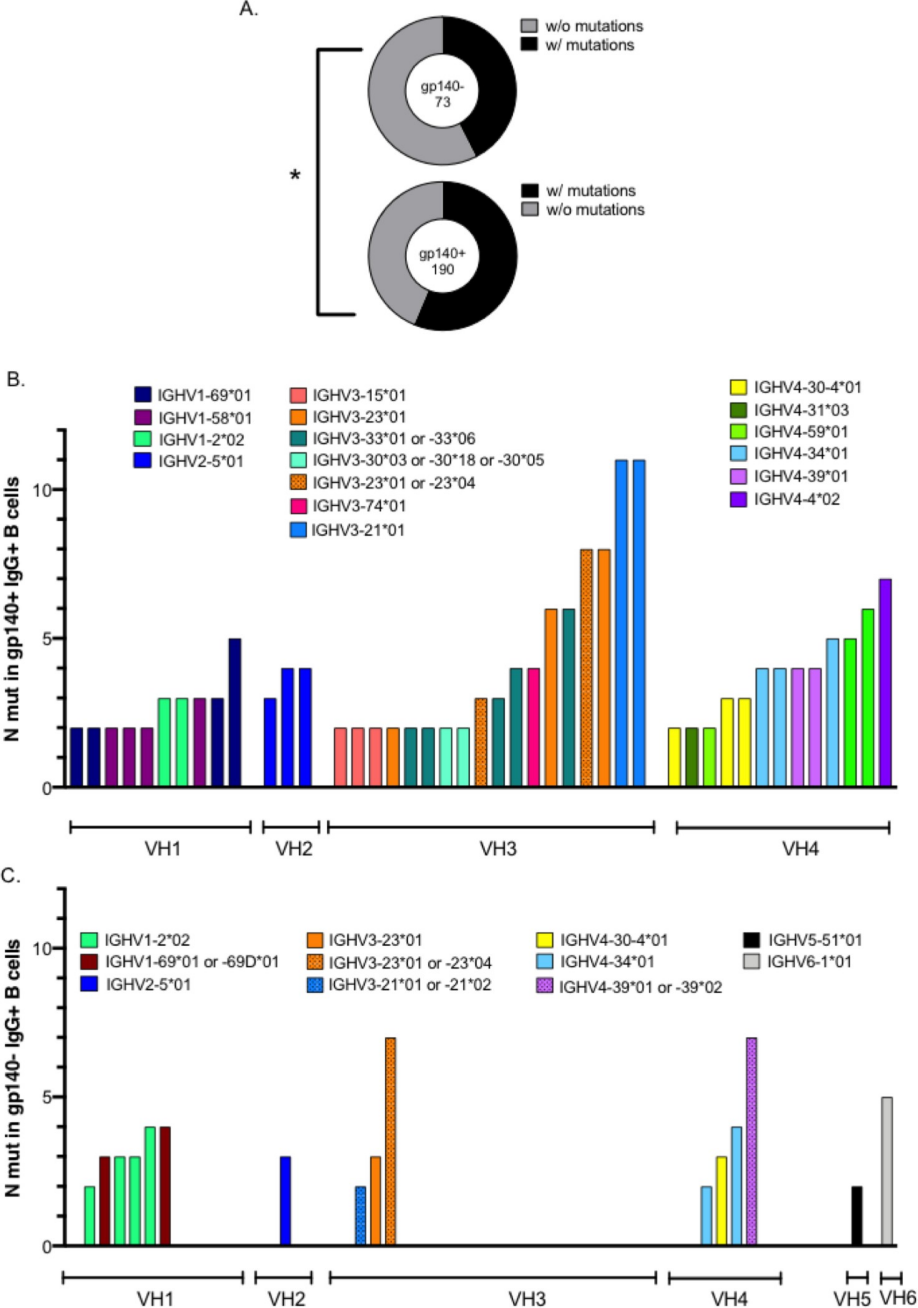
Mutated immunoglobulin VH gene status of human gp140^+^ and gp140^-^ IgG^+^ B cells in vaccinated hu-mice. **(A)** Frequencies of human gp140^+^-specific and non-specific (gp140^-^) IgG^+^ B cells, with or without mutations. The number of analyzed human cells is indicated in the center of the pie charts. Distributions were compared using Chi-Square tests. *p < 0.05. (**B-C)** Analysis of heavy chain gene segment usage and the number of somatic mutations in VH segments, carried out using NCBI IgBLAST software (http://www.ncbi.nlm.nih.gov/igblast/), in gp140^+^ (**B**) and gp140^-^ (**C**) IgG^+^ hu-B cells from all immunized hu-mice. Only IgG^+^ B cells with more than one mutation are represented.

**Fig 7:**
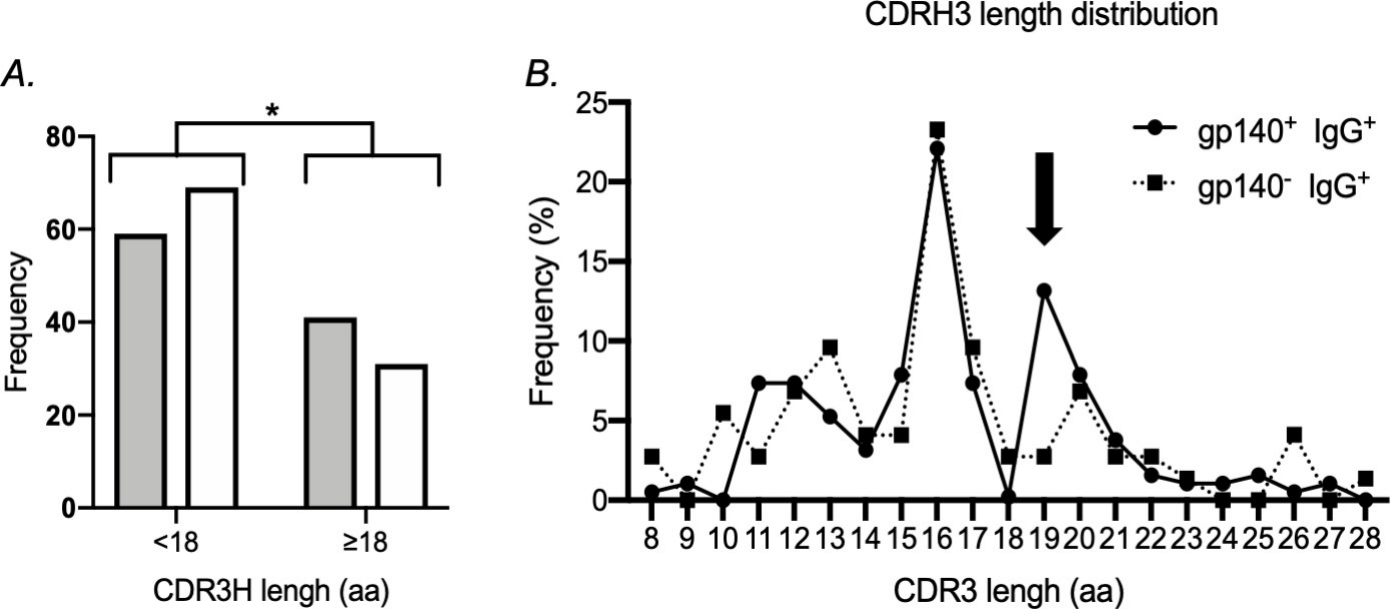
Characteristics of Complementarity Determining Region 3 of IgH (CDRH3) of human gp140^+^ and gp140^-^ IgG^+^ B cells in vaccinated hu-mice. (**A-B**) Comparison of the CDRH3 amino-acid length between gp140^+^ specific and non-specific (gp140^-^) IgG^+^ antibodies of all immunized hu-mice. (**A**) Distribution of gp140^+^ (grey bar) and gp140^-^ (white bar) IgG^+^ hu-B cells into two categories depending on whether the length of their CDRH3 was < 18 amino acids or ≥18 amino acids. Distributions were compared using Chi-Square tests. *p < 0.05. (**B**) CDRH3 length distribution in gp140^+^ (solid line) and gp140^-^ (dotted line) IgG^+^ hu-B cells of all immunized hu-mice. The arrow highlights the main difference between the two populations of IgG^+^ human B cells.

We next examined the frequency and distribution of somatic mutations of VH genes in both gp140^+^ and gp140^-^ IgG^+^ hu-B cells. The frequency of mutated clones differed significantly between these two populations, showing 56% and 42% mutated clones, with at least one mutation, in the gp140^+^ and gp140^-^ IgG^+^ hu-B cells, respectively (*P* = 0.04) (Fig 6A). The frequency of mutated sequences and distribution of somatic mutations within VH gene families are shown in Fig 6B and 6C. There was a higher frequency of mutated clones in the gp140^+^ IgG^+^ hu-B cell population, which predominated within the VH3 and VH4 families. A comparison of gp140^+^-specific VH sequences elicited by the vaccination with their respective germline sequences showed the mutations to be distributed in the (CDR)1 and CDR2 regions and the three framework regions (S7 Fig). Similarly, there was a higher rate of somatic hypermutation of Ig VL genes from the gp140^+^ IgG^+^ hu-B cells than those that were non-specific (S8 Fig). Analyses of IgH CDR3 (CDRH3) lengths showed the gp140^+^ IgG^+^ hu-B cells to exhibit a trend of a longer CDRH3 region than that of non-specific IgG^+^ hu-B cells (Fig 7A and 7B), with no increased frequency of positively charged amino acids (S9 Fig). It has been shown that length variation of CDRH3 can contribute to increased antibody specificity and influence the shape of the antibody combining sites [29,30].

### Heterologous prime/boost NC/CD vaccination increases the diversity of human B-cell clonal families

**Fig 8:**
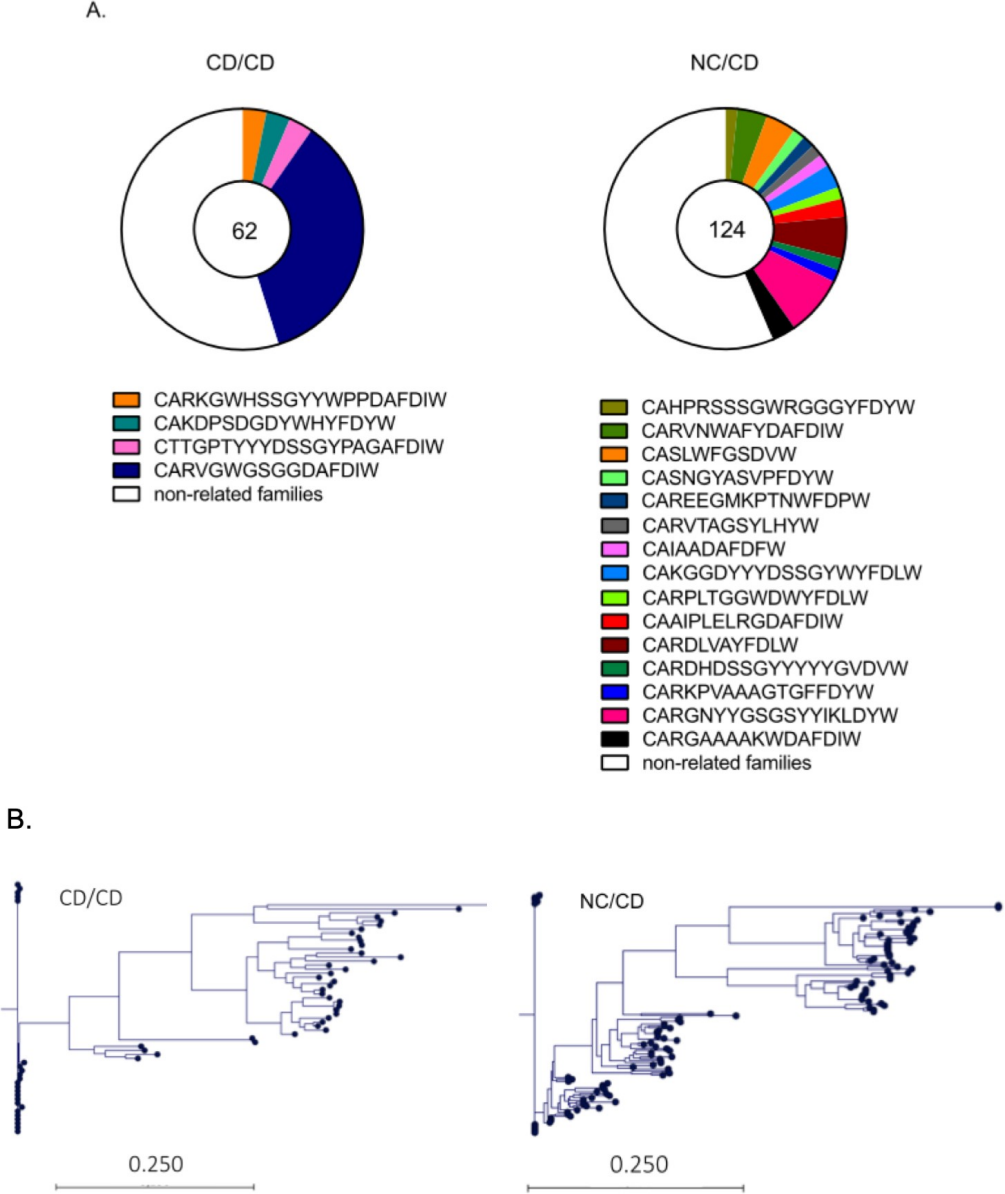
Antibody clonal families and their phylogeny within gp140^+^-specific IgG^+^ hu-B cells. **(A)** Pie charts of clonal family representation were constructed from the IgH sequences and CDR3 segment of sorted gp140^+^-specific IgG^+^ hu-B cells. The white slice represents sequences that appeared only once. The colored slices represent clonally related sequences, for which each color accounts for a different IgH clone. The numbers in the pie charts represent the number of mAbs evaluated. Human B cells were considered to belong to the same clone on the basis of identical V, D, and J gene segment usage and CDR3 length for both heavy- and light-chain Ig genes. (**B)** Phylogenic trees were generated after sequence alignment using CLC Main Workbench 7 software (v7.0) with default parameters. The relationship between sequences was determined via the neighbor-joining method. Each tip represents a BCR heavy chain sequence. The scale bar represents the genetic distance (expected changes per nucleotide site).

We then examined the number of Env-specific antibody clones elicited by the vaccine and their expansion for each vaccination regimen. The clonal diversity of gp140^+^ IgG^+^ hu-B cells was higher in hu-mice from the NC/CD prime/boost group than those of the homologous CD/CD prime/boost group by CDRH3 gene sequencing (Fig 8A). These results are consistent with the higher number of somatic hypermutations observed in the Ig VH sequences of gp140^+^ hu-B cells coming from the NC/CD group relative to those isolated from the CD/CD group (somatic hypermutation rates ranging from 0 to 11 (with 47% mutated gp140^+^ hu-B cells) and 0 to 8 (with 39% of mutated gp140^+^ hu-B cells), respectively; *P* = 0.004, Student’s t-test with Welch’s correction). The phylogenetic tree generated from IgH sequences of the gp140^+^ IgG^+^ hu-B cells is shown in Fig 8B. Analysis of the most recent common ancestry and monophyletic groups suggested the clonal evolution of specific human B cells elicited by the vaccination regimens, with the heterologous prime/boost regimen driving more diverse clonal evolution (Fig 8B).

## Discussion

After more than three decades of intensive research, a highly efficacious vaccine that can prevent HIV-1 infection is still elusive, in part due to the lack of robust preclinical models to predict HIV-1 vaccine efficacy in humans. Various prophylactic HIV-1 vaccines have been developed that elicit varying degrees of protective responses in NHP models. Some have been tested in Phase 2b/3 clinical trials and have taught us the usefulness of HIV-1 Env proteins for protective vaccine design, the need to induce both T- and B-cell responses, and the contribution of non-neutralizing antibodies directed against the HIV-1 Env V1V2 region in the prevention of HIV-1 infection [10–12,14]. However, the recent negative and disappointing results of the large Phase 3 HVTN 702 prophylactic trial put into question strategies aimed to elicit non-neutralizing anti-HIV antibodies and underscore the need to develop models for selecting candidate vaccines [31]. Although SIV challenge studies in NHPs are useful indicators for the advancement of vaccine candidates into clinical trials, they do not necessarily predict HIV-1 vaccine efficacy in humans [32]. These data highlight the need for better high-fidelity animal models for HIV-1 vaccine testing.

Among the various strategies used to develop new HIV-1 vaccines, the targeting of DCs shows promise [33,34]. Such strategies allow the delivery of minute amounts of antigen directly to DCs and are expected to more efficiently induce immune responses. Following extensive *in vitro* and *in vivo* studies, the CD40 receptor was selected as a target to develop recombinant prototype Env-targeting vaccines, due to its crucial participation in DC-T cell and DC-B cell crosstalk [35]. We have provided evidence supporting the induction of both T- and B-cell responses using an anti-CD40 monoclonal antibody linked to HIV-1 antigens via its Fc domains [16,18,21].

Here, we investigated the contribution of Tfh cells in B-cell activation and Ig repertoire diversification using a hu-mouse model immunized with the anti-CD40.Env gp140 candidate vaccine, either with a homologous prime/boost regimen or a heterologous regimen, boosting immunity primed by the NYVAC-KC vaccine. We found that hu-mice vaccinated with the anti-CD40.Env gp140 vaccine, in the presence of CpG-B as an adjuvant, developed a diverse antibody-producing hu-B cell repertoire and had circulating Env-specific IgG-switched memory human B cells that exhibit clear signs of antigen-driven antibody affinity maturation. In addition, the induction of Env-specific IgG^+^ hu-B cells significantly correlated with the emergence of human memory Tfh cells. Furthermore, the heterologous NYVAC-KC prime/anti-CD40.Env gp140 boost vaccination strategy evoked superior Env-specific humoral B-cell responses in terms of the magnitude of human B-cell responses and the clonal diversity of Env-specific human antibodies relative to the homologous CD40.Env gp140 regimen. We also showed that hu-mice, despite their relative limitations, can be useful as a pre-clinical model to rank vaccination strategies. Indeed, one striking finding of our study is that anti-CD40 targeting combined with CpG adjuvant overcomes the usual defect of human B-cell switch and maturation in hu-mice models. Several previous *in vitro* and *in vivo* studies [36,37] have demonstrated that CD40 is involved in the activation and maturation pathway of B cells. Therefore, our data suggests that, aside from targeting HIV antigens to CD40 expressed by human DCs, activation through surface CD40 molecules in the presence of CpG-B led to the maturation of naive human B cells in this hu-mouse model.

We chose to administer the anti-CD40.Env gp140 vaccine with CpG-B as an adjuvant based on our previous report that aimed to evaluate the *in vivo* impact of TLR agonists on the human immune system using hu-mouse models [22]. CpG-B is a TLR-9 agonist expressed in human plasmacytoid DCs and B cells [38,39]. One attractive property of CpG-B is its ability to trigger the secretion of type-1 IFN by plasmacytoid DCs, a cytokine involved in the differentiation of B cells into antibody-secreting B cells [38,40]. Indeed, the concomitant activation of plasmacytoid DCs through TLR9 and CD40 causes the release of high amounts of type-1 IFN as well as IL-12 [41]. Soon after the injection of CpG-B, we detected significant amounts of type-1 IFN, IL-12, and IL-6 in the blood of hu-mice [22]. These cytokines also support the maturation of conventional human DCs, which have been identified as an essential component of pre-Tfh activation [22]. We confirmed and extended this finding in the present study, with the observation of sustained upregulation of CD40 expression on conventional DCs when CpG-B was administered with the anti-CD40.Env gp140 vaccine. The use of CpG-B as an adjuvant in preventive HIV-1 vaccination can thus provide a supportive environment for the activation and differentiation of B cells into IgG-switched memory B cells. CpG-B is also considered to be a potent B-cell mitogen [42]. Naïve and memory B cells respond differently to CpG-B. In particular, naïve human B cells can proliferate and differentiate into antibody-secreting cells in response to CpG-B, but only if their BCRs are simultaneously triggered by cognate antigens [42]. In this context, we report here that hu-mice injected with CpG-B alone showed no activation of human B cells or expansion of switched memory hu-B cells, in contrast to animals immunized with the anti-CD40.Env gp140 vaccine plus CpG-B.

Our single-cell RNAseq analysis of Env-specific IgG^+^ hu-B cells generated in the vaccinated hu-mice and their comparison with non-specific IgG^+^ hu-B cells showed the anti-CD40.Env gp140 vaccine to favor an IgG3 class switch in B cells at the expense of IgG1. We have previously shown that Env-specific antibodies elicited by the anti-CD40.Env gp140 vaccine specifically recognize the V1V2 region of Env and exhibit potent ADCC properties in NHP models [16]. Indeed, in the RV144 clinical trial, the Env-IgG3 elicited by ALVAC vector prime/gp120 protein boost vaccination had ADCC functions that correlated with vaccine protection [10].

Another exciting characteristic of the Env-specific hu-B cells elicited in hu-mice by the anti-CD40.Env gp140 vaccine is the preferential use of VH3 and VH4 family genes. In addition, the VH3 and VH4 family genes displayed the highest rates of somatic hypermutations relative to those of the non-specific IgG^+^ hu-B cells used as controls. VH3 antibodies are essential for defense against a variety of viral pathogens [43,44]. Studies of human anti-HIV-1 monoclonal antibodies and VH gene usage during natural HIV-1 infection show a reduced representation of the VH3 gene family in the repertoire of anti-Env memory IgG^+^ B cells, whereas the naive VH3 IgM and IgD repertoires are relatively intact [45–47]. The reduction of VH3-expressing B cells may thus contribute to HIV-1-associated humoral immune dysfunction. Our results in hu-mice suggest that the anti-CD40.Env gp140 vaccine elicits VH3-expressing B cells with high rates of somatic hypermutations and provide further evidence supporting a pre-clinical test of the vaccine in human subjects.

Tfh cells are key partners in B-cell activation and differentiation. They are also essential for isotype switching and the selection of both high-affinity memory B-cell clones undergoing somatic hypermutation in GCs and plasma cells [48]. Tfh cell precursors, as well as mature Tfh cells in GCs, can exit lymphoid organs into the blood circulation [24,25]. These circulating CXCR5^+^ Tfh cells do not express BCL6 and are composed of distinct subsets. Among them, ICOS^+^ peripheral Tfh cells play a central role in the generation of antibody responses after influenza vaccination, likely before they exit the lymphoid organs [26]. Consistent with such a role in influenza vaccination, we showed the emergence of circulating ICOS^+^ memory hu-Tfh cells in vaccinated hu-mice that significantly correlated with the magnitude of the Env-specific hu-B cell responses elicited by the vaccine. The emergence of memory Tfh cells in the blood of vaccinated hu-mice was consistent with the induction of PD-1 and BCL6-expressing human cells inside splenic GC-like structures. Furthermore, the increased proportion of circulating ICOS^+^ human Tfh cells at day 7 post boost correlated with the increase of Env-specific IgG^+^ hu-B cells at termination. These results suggest that preventive CD40 targeting Env vaccination induces ICOS^+^ peripheral human Tfh cells and the contribution of this subpopulation of Tfh cells in the generation of Env-specific human B-cell responses. We also believe that monitoring ICOS^+^ circulating Tfh cells seven days after the last booster immunization could be considered in future studies as a surrogate marker for vaccine-induced HIV-1-specific B-cell responses, at least in hu-mice models.

Our comparative analysis between the homologous and heterologous vaccine strategies in hu-mice revealed that the anti-CD40.Env gp140 vaccine elicits a higher number of Env-specific IgG^+^ hu-B cells when used to boost NYVAC-KC-primed immune responses. In this setting, Env-specific IgG^+^ hu-B cells displayed higher numbers of somatic hypermutations. Moreover, the superior B-cell response observed in the heterologous prime/boost vaccination was associated with the emergence of a higher number of human memory ICOS^+^ Tfh cells. Consistent with these results, we showed the clonal evolution driven by the heterologous prime/boost vaccination to be more diverse. These results suggest that the anti-CD40.Env gp140 vaccine should be preferentially used to boost viral vector-primed immunity to induce a large magnitude of high-diversity Env-specific IgG^+^ memory human B-cell responses.

## Materials and methods

### Ethics statement

Human fetal liver and thymus tissues (gestational age of 16 to 20 weeks) were obtained from elective or medically indicated termination of pregnancy through a non-profit intermediary working with outpatient clinics (Advanced Bioscience Resources, Alameda, CA). Informed consent of the maternal donors was obtained in all cases under regulations governing the clinic. The project was reviewed by the University Office of Human Research Ethics, which determined that this submission did not constitute research on human subjects as defined under federal regulations [45 CFR 46.102 (d or f) and 21 CFR 56.102(c)(e)(l)]. All animal studies were approved by the University of North Carolina Institutional Animal Care and Use Committee (IACUC ID 14–100).

### Generation of hu-mice

NRG (NOD-Rag2^–/–^γc^–/–^) mice were purchased from The Jackson Laboratory and maintained in a specific pathogen-free environment. Hu-mice were generated as previously described [21]. Briefly, six- to eight-week-old NRG mice were sub-lethally irradiated. CD34^+^ hematopoietic progenitor cells purified from human fetal liver were then injected intravenously. Human immune-cell engraftment was verified by flow cytometry 12 weeks after transplantation (S2 Fig). We used different human donors for different cohorts of hu-mice in the study.

### Production of anti-CD40.Env gp140 vaccine and vaccination regimens

Recombinant anti-human CD40 antibody fused to the gp140ZM96 Clade C protein (anti-CD40.Env gp140 vaccine) was produced as previously described [8,16], except that we humanized the mouse variable regions to reduce antigenicity in humans. The gp140ZM96 Clade C sequence was derived from the codon optimized HIV-1 96ZM651 synthetic construct (NIH AIDS reagent program, GenBank AY181197.1 residues 94–2064) as described in [8,16]. The vaccine had low lipopolysaccharide levels of 0.04 ng/mg protein. The construct, NYVAC-KC-expressing gp140ZM96 Clade C (Nyvac-KC-gp140ZM96), is a replication-competent, attenuated recombinant of the vaccinia virus strain NYVAC described in previous reports [15,49]. For vaccination, hu-mice were intraperitoneally co-injected with 10 μg anti-CD40.Env gp140 and 50 μg CpG-B (Sigma) or 2 x 10^7^ PFU NYVAC-KC- gp140ZM96 with 50 μg CpG-B. Control hu-mice received PBS or CpG-B treatment only.

### Flow cytometry

For surface staining, single-cell suspensions prepared from the spleens of hu-mice or total blood cells collected in EDTA-coated tubes were stained for surface markers and analyzed on a BD LSRFortessa Beckton-Dickinson). Human CD11c-PacBlue, CD45-AF700, CD45-APC-Cy7, HLA-DR-PeCy7, CD40-PeCF, CD19-PeCy5, CD19-APC, CD19-PacBlue, CD27-APC, IgG-PeCy5, IgG-PercpCy5.5, IgD-FITC, IgM-FITC, CD3-APC-H7, CD3-FITC, CD14-FITC, CXCR5-AF488, and streptavidin-PE were purchased from Becton-Dickinson, whereas human CCR7-PacBlue, CD45RA-FITC, IgM-PacBlue, and ICOS-APC were purchased from Biolegend. Pacific orange-conjugated anti-mouse CD45, PE/Texas red-conjugated anti-human CD4, and the LIVE/DEAD Fixable Aqua (LD7) Dead Cell Stain Kit were purchased from Invitrogen. Data were analyzed using FlowJo software (FlowJo, LLC).

### Luminex binding assay

Gp120 (clade C), gp140ZM96 (clade C) and BG505 SOSIP gp140 (clade A), proteins were conjugated to Bio-Plex magnetic beads (Bio-Rad) according to the manufacturer’s protocol and used in a primary screen for anti–Env human Ab binding studies. After a step of serial dilutions, plasmas from hu-mice were incubated with Env-coated beads for 1 h, beads were washed with PBS and bound human Ab detected using a PE-labeled anti-human IgG secondary (OneLambda LS-AB2) in a Luminex binding assay. Beads were analyzed on a Luminex 200 instrument (Luminex Corporation). Results are expressed as MFI signal relative to negative control.

### Gp140ZM96-specific IgG^+^ human B cell sorting

Frozen spleen-cell suspensions were thawed at 37°C in RPMI plus 10% fetal calf serum (FCS) and washed and resuspended in flow-cytometry buffer (2% FCS in PBS). Cells were first incubated with 1 μg recombinant gp140ZM96-biotinylated protein for 20 min on ice as previously described [50]. The cells were then washed in flow-cytometry buffer and incubated with a mix of fluorophore-conjugated antibodies (-mCD45-APC-Cy7/hCD19-APC/hCD14-FITC/hCD3-FITC/hIgM-FITC/hIgG-PercpCy5.5) and PE-streptavidin (from BD) for 30 min on ice. Cells were washed again and then incubated with Live/Dead Fixable Aqua Dead Cell Stain (Thermofisher) for 10 min on ice. After a final wash in flow-cytometry buffer, cells were resuspended in flow-cytometry buffer at a concentration of 1 × 10^7^ cells per mL for single cell sorting on a BD FACSAria (BD Biosciences).

### Integrative single-cell analysis

First, gp140^+^ and gp140^-^ IgG^+^ human B cells were sorted on a BD FACSAriaII, one cell per well, in 96-well plates containing specific FB5P-seq lysis buffer [28] at the Cybio Facility (Cochin Institute, Paris). Plates were immediately frozen for storage at -80°C and sent on dry ice to the Genomics Core Facility of the CIML, Marseille, for generating scRNAseq libraries with the FB5P-seq protocol as we have previously described [28]. Briefly, mRNA reverse transcription (RT), cDNA 5’-end barcoding, and PCR amplification were performed using a template switching (TS) approach. After amplification, barcoded full-length cDNA from each well was pooled for purification and library preparation. An Illumina sequencing library targeting the 5’-end of barcoded cDNA was prepared for each plate by a modified transposase-based method incorporating a plate-associated i7 barcode. Resulting libraries had a broad size distribution, resulting in gene template reads covering the 5’-end of transcripts from the 3^rd^ to the 60^th^ percentile of gene body length, on average. As a consequence, sequencing reads covered the entire variable region and a significant portion of the constant region of the IGH and IGK or IGL expressed mRNAs, enabling assembly and reconstitution of the BCR repertoire from scRNAseq data.

Libraries prepared with the FB5P-seq protocol were sequenced on the Illumina NextSeq550 platform with High-Output 75-cycle flow cells, targeting 5x10^5^ reads per cell in paired-end single-index mode with the following configuration: Read1 (gene template) 67 cycles, Read i7 (plate barcode) 8 cycles, and Read2 (cell barcode and Unique Molecular Identifier) 16 cycles.

We used a custom bioinformatics pipeline to process fastq files and generate single-cell gene expression matrices and BCR sequence files, as we have previously described [28]. Briefly, the pipeline to obtain gene expression matrices was adapted from the Drop-seq pipeline [51] and relied on extracting the cell barcode and UMI from Read2 and aligning Read1 on the reference genome using STAR and HTSeqCount. For BCR sequence reconstruction, we used Trinity for *de novo* transcriptome assembly for each cell based on Read1 sequences and then filtered the resulting isoforms for productive BCR sequences using MigMap, BlastN, and Kallisto. Briefly, MigMap was used to assess whether reconstructed contigs corresponded to a productive V(D)J rearrangement and to identify germline V, D, and J genes and CDR3 sequence for each contig. For each cell, reconstructed contigs corresponding to the same V(D)J rearrangement were merged, keeping the largest sequence for further analysis. We used BlastN to align the reconstructed BCR contigs against reference sequences of constant region genes, and discarded contigs with no constant region identified in-frame with the V(D)J rearrangement. Finally, we used the pseudo-aligner Kallisto to map each cell’s FB5P-seq Read1 sequences on its reconstructed contigs and quantify contig expression. If several contigs corresponding to the same BCR chain passed the above filters, we retained the contig with the highest level of expression.

Following *in silico* reconstruction of BCR sequences from scRNAseq reads, nucleotide sequences were submitted to IMGT HighV-QUEST analysis [52] to identify V and J genes, CDR3 junction sequences, and other BCR sequence features. We further processed the IMGT HighV-QUEST output summary tables in Microsoft Excel.

Quality control was performed on scRNAseq data to remove poor quality cells. Cells with less than 250 detected genes were removed. We further excluded cells with values below three median absolute deviations (MADs) from the median for UMI counts, the number of genes detected, or ERCC accuracy and cells with values above three MADs from the median for ERCC transcript percentage, as we have previously described [28]. For each cell that passed the quality control, gene expression UMI count values were log normalized using Seurat v3.0.0.9 *NormalizeData* [53] with a scale factor of 10,000. The resulting datasets were then exported as comma-separated value files for further analysis with SeqGeq v1.6 (BD FlowJo) for Windows 10. For the final analysis, cells with fewer than 500 detected genes or more than 7.5% mitochondrial genes among expressed genes were excluded and principal component analysis performed followed by tSNE embedding (based on 10 principal components) on the remaining cells (n = 783). Only cells with a detected IGHG1, IGHG2, IGHG3, or IGHG4 containing a productive IGH rearrangement were conserved (n = 280) and we excluded outlier cells with gene expression profiles of myeloid cells, pre-B cells, or naïve B cells, based on the tSNE embedding and marker gene expression. The remaining high-quality cells (n = 263) were assigned to five distinct B-cell subsets based on their expression of subset-specific gene signatures (previously computed on bona fide human tonsil B cell subset scRNA-seq datasets [28]) and marker genes, as described in S3 Fig.

### Immunohistochemistry

Deparaffinized spleen tissue sections were stained with hematoxylin, eosin, and saffron for morphological evaluation and for CD20 (L26 clone, DakoCytomation, Glostrup, Denmark), CD3 (F.7.2.38 clone, DakoCytomation, Glostrup, Denmark), BCL6 (LN22 clone, Menarini-Leica, Rungis, France), and PD-1 (NAT105 clone, Abcam, Paris, France) using a BOND-III Autostainer (Leica Microsystems, Newcastle-upon-Tyne, UK). Tissue section staining was performed using the DAB Substrate after antigen retrieval by heating (pH6 for PD1 and pH9 for the other antibodies). All slides were counterstained with hematoxylin. All immuno-histochemical images were acquired on a Zeiss Axioplan 2 plus (Götttingen, Germany) microscope at x10 (0.30 NA) and x20 (0.50 NA) magnifications using a Canon EOS600D digital camera and Canon EOS utility software (Götttingen, Germany).

### Statistics

Statistical analyses were performed using GraphPad Prism 9.0 software (GraphPad Software). Individual values are presented, along with the median. Experiments were analyzed using a two-tailed Student's *t*-test or two-tailed Mann-Whitney *U*-test, or two-sided Chi-square test, according to the assumptions of the test, and as indicated in the figure legends. A *P* value < 0.05 was considered to be significant.

## Supporting information

S1 Fig Gating strategies to identify human immune cell populations in the blood of hu-mice. These staining were performed on fresh blood before hu-mouse immunization. Top panel was used to identify the human (hu) B cells, human myeloid DCs (mDCs) and human plasmacytoid DCs (pDCs) as well as human monocytes. After gating for single cells, viable cells within the huCD45^+^ mouse (m)CD45^-^ gate were represented in a huCD19 *versus* huCD11c dot blot to identify hu-B cells and hu-mDCs. Human monocytes and hu-pDCs were selected on a huCD14 versus huBDCA2 dot blot within the CD19^-^ CD11c^-^ population. Lower panel was used to identify huCD4^+^ and CD8^+^ T cells. After gating for single cells, viable cells within the huCD45^+^ mCD45^-^ gate were represented in a huCD3 *versus* huCD19 dot blot. Hu-CD4^+^ and CD8^+^ T cells were further selected on the huCD3 *versus* huCD4 dot blot. The gating strategy described in the lower panel was also applied in the hu-mouse spleens to identify hu-B cells, hu-T cells and hu-CD4^+^ T cells.
(TIFF)

S2 Fig Human stem-cell reconstitution of NRG hu-mice. (**A**) Frequencies of human monocytes, human plasmacytoid (p) and myeloid (m) DCs, hu- B and hu-T lymphocytes in the blood of NRG hu-mice at baseline. Individual values are presented. (**B**) Frequencies of hu-CD45^+^ cells in the blood of NRG hu-mice at baseline and week 6 (one week after the last immunization). Individual values are presented. (**C**) Absolute number of human CD45^+^ cells in the spleen of hu-mice at week 6. Individual values are presented, along with the median. Two-sided Mann-Whitney U-tests were used for comparisons between immunized and non-immunized hu-mice. *p < 0.05.
(TIFF)

S3 Fig Vaccination elicits expansion of human memory B cells. **(A)** Gating strategy for the identification of human memory-switched B cells. Flow cytometry of splenocytes from hu-mice injected three times with the anti-CD40.Env gp140 vaccine (CD/CD), one week after the last injection. The human CD19^+^ B cells (see their gating strategy in the S1 Fig) were represented in a huIgD *versus* huCD27 dot blot to identify the IgD^-^/CD27^+^ human memory B cells. Then the total IgG-switched hu-B cells was assessed in CD27^+^ memory hu-B cell subsets. **(B-C)** Total IgG-switched CD27^+^ memory hu-B cells assessed in the blood (**B)** and spleens **(C)** of hu-mice. Individual values are presented, along with the median. Two-sided Mann-Whitney U-tests were used for comparisons between immunized and non-immunized hu-mice. *p < 0.05, **p<0.01.
(TIFF)

S4 Fig Evaluation of specific humoral immune responses elicited by the IgG4-gp140ZM96 control construct. Frequency of gp140-specific IgG^+^ hu-B cells at w6 in the blood of hu-mice immunized with the IgG4-gp140ZM96 plus CpG, the αCD40.Env gp140 vaccine (CD/CD) or control hu-mice injected with CpG (n = 3) or PBS (n = 2). Individual values are presented, along with the median. Two-sided Mann-Whitney *U*-test was used for the comparison. *p<0.05.
(TIFF)

S5 Fig Gating strategy for sorting human gp140^+^ and gp140^-^ Ig^+^ B cells. Whole spleen cells from immunized hu-mice were stained and used for the single cell sorting of human gp140^+^ and gp140^-^ Ig^+^ B cells. (**A**) Gating strategy shown on a fraction of whole cells recorded before starting the single cell sort. After gating for single cells, viable human cells within the mCD45^-^ cells were represented in a huCD3/CD14/IgM *versus* huCD19 dot blot to select the huB cells. Then, hu-B cells were represented on a gp140ZM96 *versus* huIgG dot blot. For a matter of limited number of specific cells, we did not record enough IgG/gp140 cells to show a picture before the sort for keeping the maximum number of cells for single cell sorting. (**B**) The dot blot analyses show concatenated data from all collected hu-B cells represented either in a gp140ZM96 *versus* hu-IgG dot blot or a gp140ZM96 *versus* hu-CD19 dot blot.
(TIFF)

S6 Fig Distribution of human B-cell subsets among the gp140^+^ and gp140^-^ IgG^+^ hu-B cells. **(A-C)** Single (CD3/CD14/IgM/Vivid/mCD45)^-^ CD19^+^ IgG^+^ gp140^+^ or gp140^-^ huB cells were identified among the spleen cells of mice receiving the CD/CD or NC/CD vaccines, single-cell sorted into 96-well PCR plates, and subjected to scRNA-seq (see Methods). (**A**) Identification of IgG^+^ B-cell subsets based on the single-cell expression of subset-specific signatures, enabling the discrimination of plasma cells and plasmablasts from non-antibody producing B cells (left), and memory, activated memory (Act. Mem.), and GC B cells within non-antibody-producing B cells (right). (**B**) Gene expression heatmap of human IgG^+^ Memory, Activated Memory (Act. Mem.), GC, Plasmablasts, and Plasma cells for the indicated marker genes. (**C**) Distribution of Memory, Act. Mem., and GC B cells, Plasmablasts, and Plasma cells among the gp140^+^ and gp140^-^ IgG^+^ hu-B cells sorted from all immunized hu-mice. The number of B cells analyzed is indicated in the center of each pie chart.
(TIFF)

S7 Fig Position of FWR and CDR mutations in the IgH from gp140^+^-specific human B cells. Analysis of heavy-chain gene-segment usage, the number of somatic mutations, and their position in the FWR and CDR regions was carried out using NCBI IgBLAST software (http://www.ncbi.nlm.nih.gov/igblast/). CDRs and FWRs were assigned according to the IMGT numbering system using IgBLAST software. Alignment of VH amino-acid sequences from anti-gp140 monoclonal antibodies of the same VH class carrying near-identical CDRH3s. Amino acids that differ from the common germline VH are indicated and identical residues are denoted by a dash.
(TIFF)

S8 Fig Mutated immunoglobulin variable light-chain gene status. Violin plots comparing the number of mutations (mut.) in the variable genes of light (IgL) chains between gp140+ and gp140- IgG+ hu-B cells isolated from all immunized hu-mice. Black solid lines indicate the median. The number of sequences analyzed (n) in each group of human B cells is indicated along the x-axis. Groups were compared using Student’s unpaired one-sided t-test with Welch’s correction. *p < 0.05.
(TIFF)

S9 Fig Comparison of the number of positive charges in the CDRH3 between gp140^+^ and gp140^-^ IgG^+^ antibodies isolated from all immunized hu-mice. Distribution of gp140^+^ and gp140^-^ IgG^+^ hu-B cells into two categories depending on whether the number of positive amino acids in their CDRH3 is between 0 and 3 or ≥ 4 amino acids. The two-sided Chi-Square test was used to compare distributions. ^ns^p > 0.05.
(TIFF)
