# Supplementary figures and images for: TLR-9 agonist and CD40-targeting vaccination induces HIV-1 envelope-specific B cells with a diversified immunoglobulin repertoire in humanized mice
Source: PLoS Pathog. 2020 Nov 30;16(11):e1009025. doi: 10.1371/journal.ppat.1009025 (PMC7728200; doi:10.1371/journal.ppat.1009025)

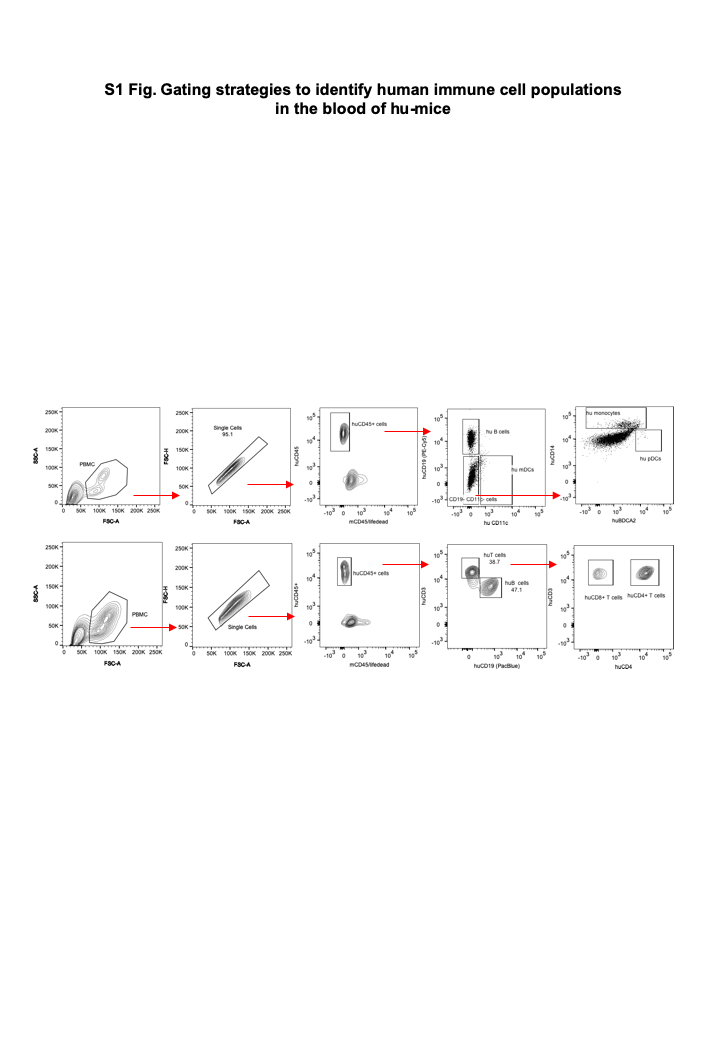

Supplement: S1 Fig — These staining were performed on fresh blood before hu-mouse immunization. Top panel was used to identify the human (hu) B cells, human myeloid DCs (mDCs) and human plasmacytoid DCs (pDCs) as well as human monocytes. After gating for single cells, viable cells within the huCD45+ mouse (m)CD45- gate were represented in a huCD19 versus huCD11c dot blot to identify hu-B cells and hu-mDCs. Human monocytes and hu-pDCs were selected on a huCD14 versus huBDCA2 dot blot within the CD19- CD11c- population. Lower panel was used to identify huCD4+ and CD8+ T cells. After gating for single cells, viable cells within the huCD45+ mCD45- gate were represented in a huCD3 versus huCD19 dot blot. Hu-CD4+ and CD8+ T cells were further selected on the huCD3 versus huCD4 dot blot. The gating strategy described in the lower panel was also applied in the hu-mouse spleens to identify hu-B cells, hu-T cells and hu-CD4+ T cells. (TIFF) [file ppat.1009025.s001.tiff]

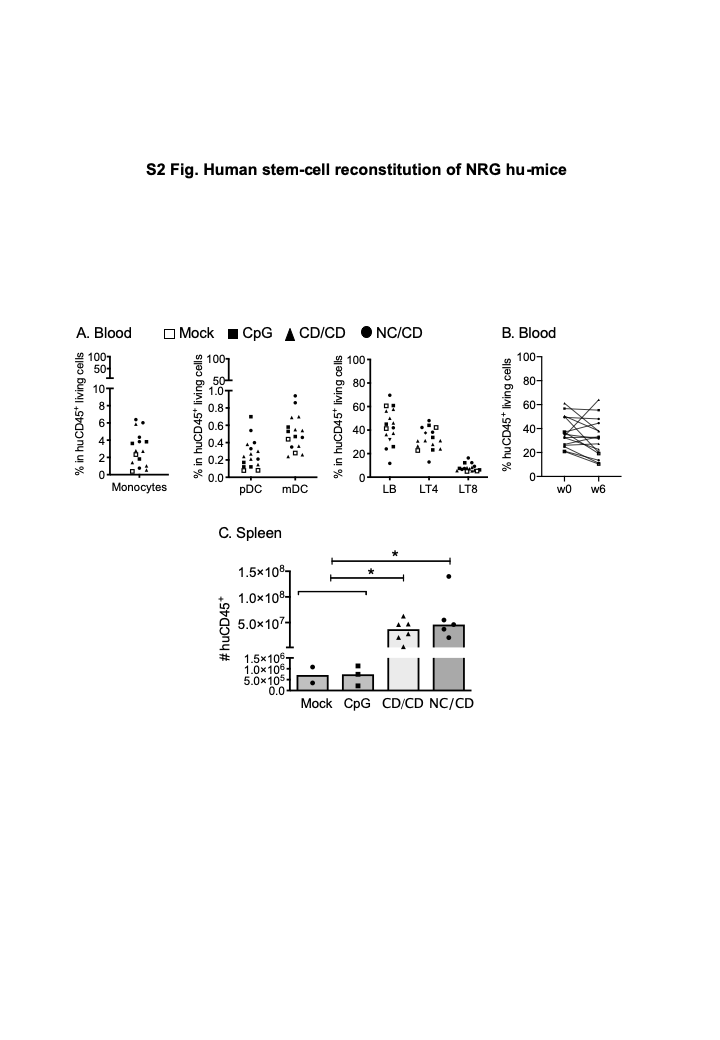

Supplement: S2 Fig — (A) Frequencies of human monocytes, human plasmacytoid (p) and myeloid (m) DCs, hu- B and hu-T lymphocytes in the blood of NRG hu-mice at baseline. Individual values are presented. (B) Frequencies of hu-CD45+ cells in the blood of NRG hu-mice at baseline and week 6 (one week after the last immunization). Individual values are presented. (C) Absolute number of human CD45+ cells in the spleen of hu-mice at week 6. Individual values are presented, along with the median. Two-sided Mann-Whitney U-tests were used for comparisons between immunized and non-immunized hu-mice. *p < 0.05. (TIFF) [file ppat.1009025.s002.tiff]

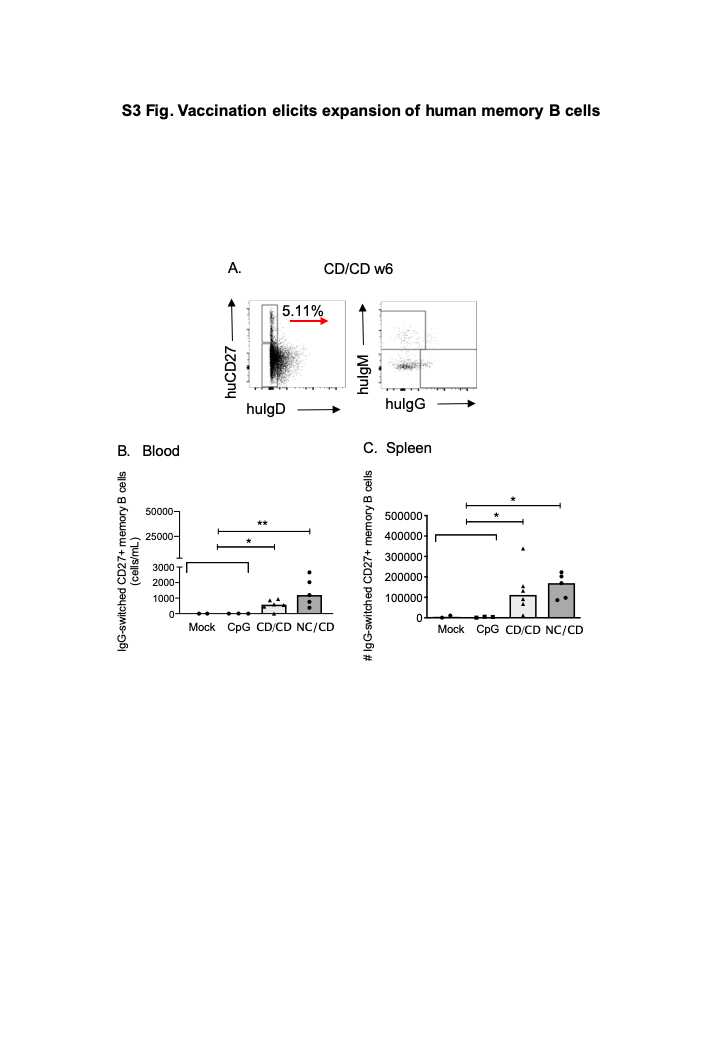

Supplement: S3 Fig — (A) Gating strategy for the identification of human memory-switched B cells. Flow cytometry of splenocytes from hu-mice injected three times with the anti-CD40.Env gp140 vaccine (CD/CD), one week after the last injection. The human CD19+ B cells (see their gating strategy in the S1 Fig) were represented in a huIgD versus huCD27 dot blot to identify the IgD-/CD27+ human memory B cells. Then the total IgG-switched hu-B cells was assessed in CD27+ memory hu-B cell subsets. (B-C) Total IgG-switched CD27+ memory hu-B cells assessed in the blood (B) and spleens (C) of hu-mice. Individual values are presented, along with the median. Two-sided Mann-Whitney U-tests were used for comparisons between immunized and non-immunized hu-mice. *p < 0.05, **p<0.01. (TIFF) [file ppat.1009025.s003.tiff]

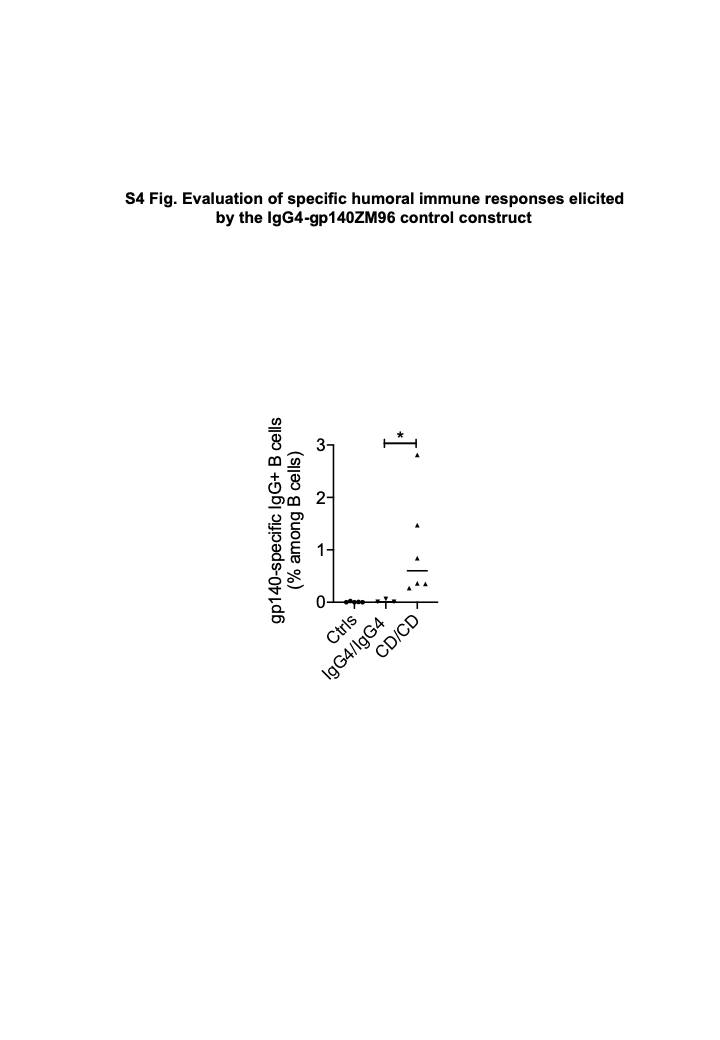

Supplement: S4 Fig — Frequency of gp140-specific IgG+ hu-B cells at w6 in the blood of hu-mice immunized with the IgG4-gp140ZM96 plus CpG, the αCD40.Env gp140 vaccine (CD/CD) or control hu-mice injected with CpG (n = 3) or PBS (n = 2). Individual values are presented, along with the median. Two-sided Mann-Whitney U-test was used for the comparison. *p<0.05. (TIFF) [file ppat.1009025.s004.tiff]

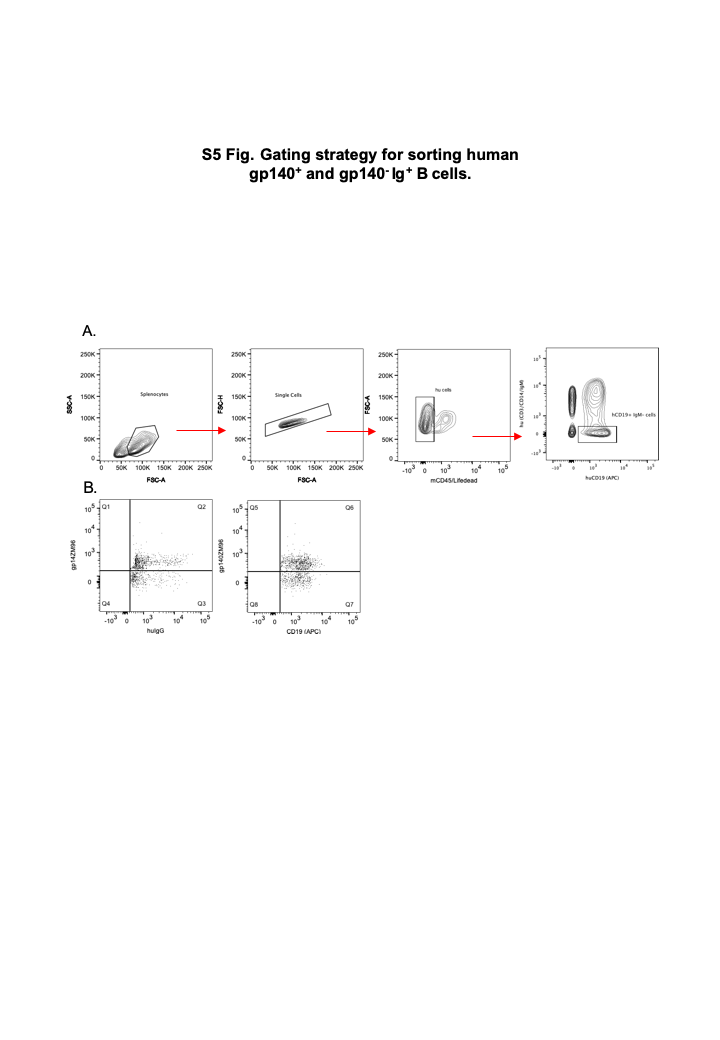

Supplement: S5 Fig — Whole spleen cells from immunized hu-mice were stained and used for the single cell sorting of human gp140+ and gp140- Ig+ B cells. (A) Gating strategy shown on a fraction of whole cells recorded before starting the single cell sort. After gating for single cells, viable human cells within the mCD45- cells were represented in a huCD3/CD14/IgM versus huCD19 dot blot to select the huB cells. Then, hu-B cells were represented on a gp140ZM96 versus huIgG dot blot. For a matter of limited number of specific cells, we did not record enough IgG/gp140 cells to show a picture before the sort for keeping the maximum number of cells for single cell sorting. (B) The dot blot analyses show concatenated data from all collected hu-B cells represented either in a gp140ZM96 versus hu-IgG dot blot or a gp140ZM96 versus hu-CD19 dot blot. (TIFF) [file ppat.1009025.s005.tiff]

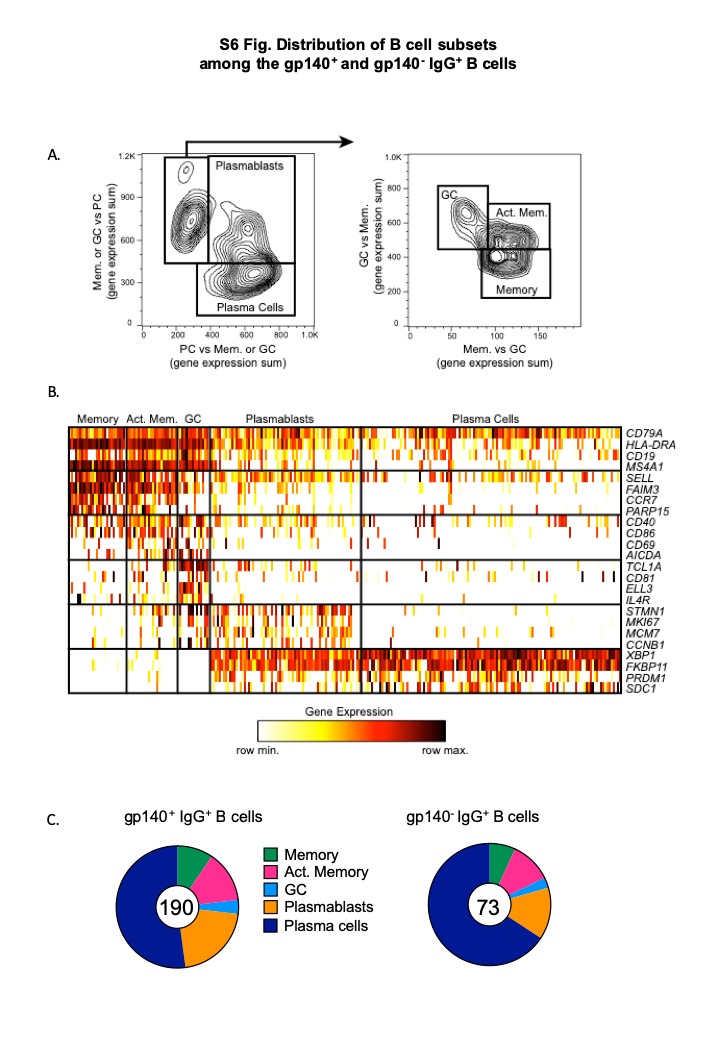

Supplement: S6 Fig — (A-C) Single (CD3/CD14/IgM/Vivid/mCD45)- CD19+ IgG+ gp140+ or gp140- huB cells were identified among the spleen cells of mice receiving the CD/CD or NC/CD vaccines, single-cell sorted into 96-well PCR plates, and subjected to scRNA-seq (see Methods). (A) Identification of IgG+ B-cell subsets based on the single-cell expression of subset-specific signatures, enabling the discrimination of plasma cells and plasmablasts from non-antibody producing B cells (left), and memory, activated memory (Act. Mem.), and GC B cells within non-antibody-producing B cells (right). (B) Gene expression heatmap of human IgG+ Memory, Activated Memory (Act. Mem.), GC, Plasmablasts, and Plasma cells for the indicated marker genes. (C) Distribution of Memory, Act. Mem., and GC B cells, Plasmablasts, and Plasma cells among the gp140+ and gp140- IgG+ hu-B cells sorted from all immunized hu-mice. The number of B cells analyzed is indicated in the center of each pie chart. (TIFF) [file ppat.1009025.s006.tiff]

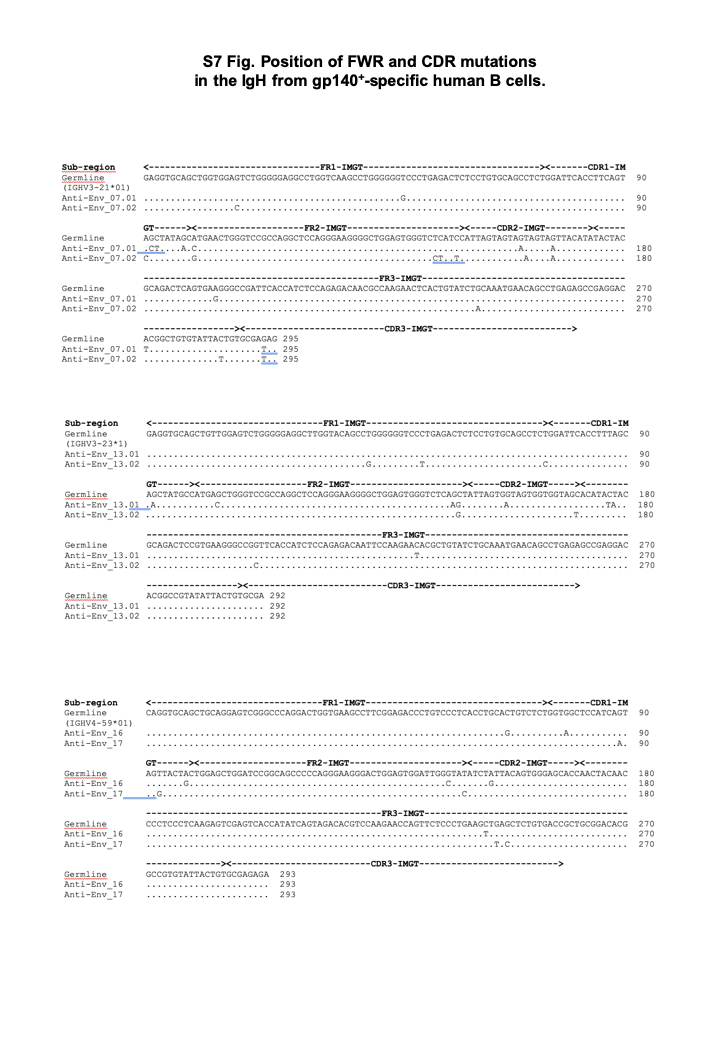

Supplement: S7 Fig — Analysis of heavy-chain gene-segment usage, the number of somatic mutations, and their position in the FWR and CDR regions was carried out using NCBI IgBLAST software (http://www.ncbi.nlm.nih.gov/igblast/). CDRs and FWRs were assigned according to the IMGT numbering system using IgBLAST software. Alignment of VH amino-acid sequences from anti-gp140 monoclonal antibodies of the same VH class carrying near-identical CDRH3s. Amino acids that differ from the common germline VH are indicated and identical residues are denoted by a dash. (TIFF) [file ppat.1009025.s007.tiff]

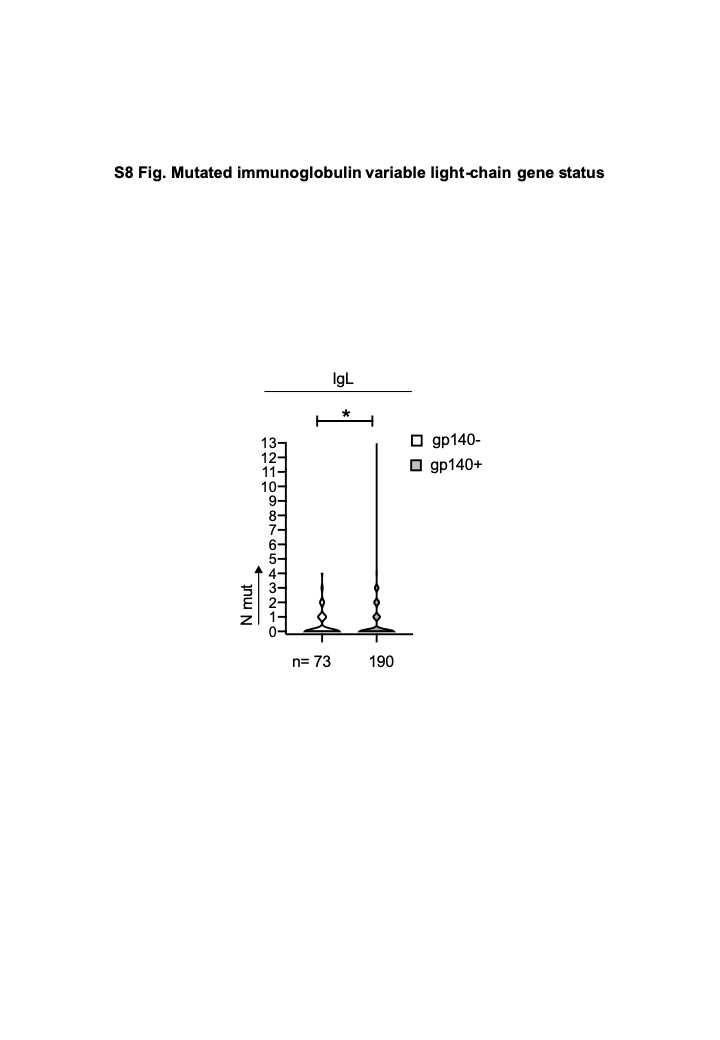

Supplement: S8 Fig — Violin plots comparing the number of mutations (mut.) in the variable genes of light (IgL) chains between gp140+ and gp140- IgG+ hu-B cells isolated from all immunized hu-mice. Black solid lines indicate the median. The number of sequences analyzed (n) in each group of human B cells is indicated along the x-axis. Groups were compared using Student’s unpaired one-sided t-test with Welch’s correction. *p < 0.05. (TIFF) [file ppat.1009025.s008.tiff]

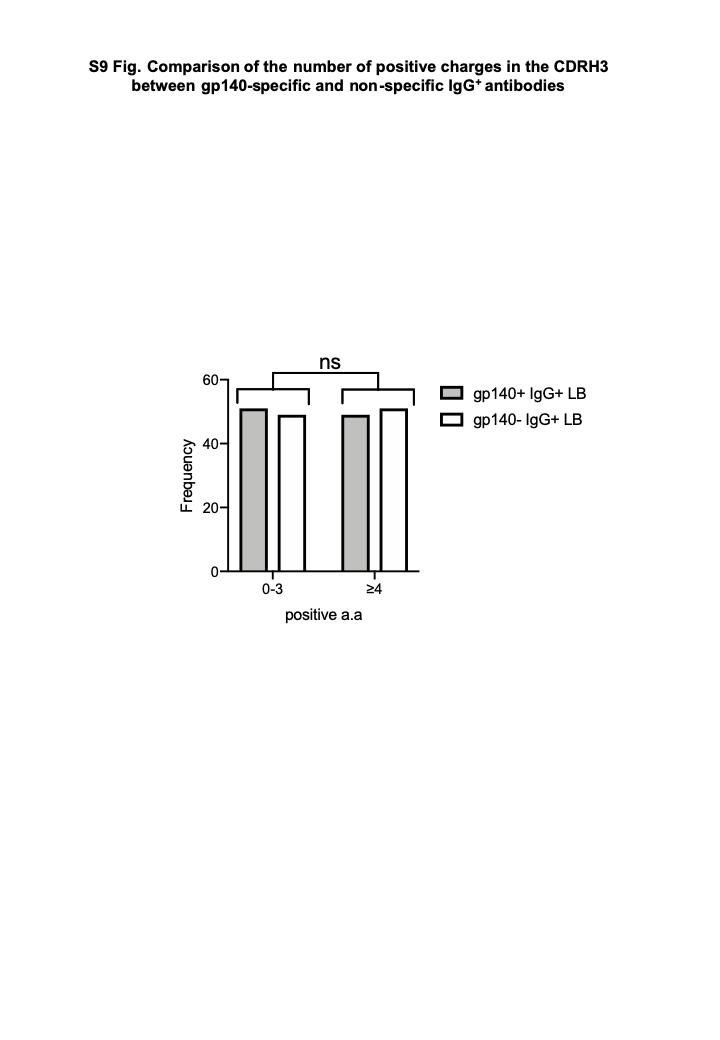

Supplement: S9 Fig — Distribution of gp140+ and gp140- IgG+ hu-B cells into two categories depending on whether the number of positive amino acids in their CDRH3 is between 0 and 3 or ≥ 4 amino acids. The two-sided Chi-Square test was used to compare distributions. nsp > 0.05. (TIFF) [file ppat.1009025.s009.tiff]
